# *Tp53* haploinsufficiency is involved in hotspot mutations and cytoskeletal remodeling in gefitinib-induced drug-resistant *EGFR*^*L858R*^-lung cancer mice

**DOI:** 10.1038/s41420-023-01393-2

**Published:** 2023-03-14

**Authors:** Yi-Shiang Wang, Ming-Jer Young, Chia-Yu Liu, Yung-Ching Chen, Jan-Jong Hung

**Affiliations:** 1 Institute of Basic Medical Sciences, National Cheng Kung University, Tainan, Taiwan; 2 Department of Biotechnology and Bioindustry Sciences, National Cheng Kung University, Tainan, Taiwan

**Keywords:** Cancer genomics, Cancer epigenetics

## Abstract

Tumor heterogeneity is the major factor for inducing drug resistance. p53 is the major defender to maintain genomic stability, which is a high proportion mutated in most of the cancer types. In this study, we established in vivo animal models of gefitinib-induced drug-resistant lung cancer containing *EGFR*^*L858R*^ and *EGFR*^L858R^*Tp53^*+/−*^ mice to explore the molecular mechanisms of drug resistance by studying the genomic integrity and global gene expression. The cellular morphology of the lung tumors between gefitinib-induced drug-resistant mice and drug-sensitive mice were very different. In addition, in drug-resistant mice, the expression of many cytoskeleton-related genes were changed, accompanied by decreased amounts of actin filaments and increased amounts of microtubule, indicating that significant cytoskeletal remodeling is induced in gefitinib-induced drug-resistant *EGFR*^*L858R*^ and *EGFR*^L858R^*Tp53^*+/−*^ lung cancer mice. The gene expression profiles and involved pathways were different in gefitinib-sensitive, gefitinib-resistant and *Tp53*^*+/−*^*-*mice. Increases in drug resistance and nuclear size (N/C ratio) were found in *EGFR*^L858R^*Tp53^*+/−*^ drug-resistant mice. Mutational hotspot regions for drug resistance via *Tp53*^*+/+*^*-* and *Tp53*^*+/−*^*-*mediated pathways are located on chromosome 1 and chromosome 11, respectively, and are related to prognosis of lung cancer cohorts. This study not only builds up a gefitinib-induced drug-resistant *EGFR*^*L858R*^ lung cancer animal model, but also provides a novel mutation profile in a *Tp53*^*+/+*^- or *Tp53*^*+/−*^-mediated manner and induced cytoskeleton remodeling during drug resistance, which could contribute to the prevention of drug resistance during cancer therapy.

## Introduction

Lung cancer including the non-small cell lung cancer (NSCLC) and small cell lung cancer (SCLC) is the leading cause of cancer-related death worldwide [1]. Approximately 85% of all new lung cancer cases are NSCLC [2], and adenocarcinoma is the most common subtype of NSCLC. Two genes, *Kras* and *EGFR*, are the most important for lung cancer formation. Many mutation sites of EGFR have been determined to be involved in lung cancer formation. Exon 19 deletion and leucine at 858^th^ changed to arginine (L858R) are the most frequently found in lung cancer patients [3]. Genomic instability is one of the most important hallmarks and is involved in nearly all other hallmarks [4]. Genomic instability results in a high frequency of mutation, including chromosomal rearrangements, aneuploidy and changes in nucleic acid sequences [5]. An increase in DNA damage caused by processes such as irradiation or a decrease in DNA repair activity promotes genomic instability [6]. Many studies have also indicated that various therapeutic methods, such as radiotherapy, chemotherapy, and targeted therapy, induce genomic instability, subsequently increasing tumor heterogeneity and ultimately leading to recurrence [7]. Understanding the mechanisms by which genomic instability can be induced and trying to inhibit these mechanisms are urgent.

Genome stabilization can decrease the rate of mutation and prevent the dysregulation of genes, potentially inhibiting drug resistance during cancer therapy [8]. Most of the previous studies on drug resistance were performed in vitro [9]. However, tumor heterogeneity is induced in vivo but not in vitro in cancer cells [10]. Mouse chromosome 11 deficiency will influence p53 tumorigenicity during cancer progression and the therapeutic period, thereby causing cancer malignancy and recurrence [11]. Most of the mutation sites are located in the DNA binding domain, thus conferring loss of the DNA binding ability and subsequent failure to be recruited to the promoters of the target genes [12]. Most p53 target genes are involved in cell cycle progression, DNA damage repair activity and apoptosis [13]. Therefore, p53 mutation induces genomic instability, subsequently increasing tumor heterogeneity [14]. Previous studies have indicated that p53 mutation during cancer progression generates a mutational hotspot on chromosome 17 that contains many oncogenes, such as *brca1* [15–17]. However, the effect of *Tp53* mutation on drug resistance is interesting and needs to be clarified. Recent studies have revealed that p53 is involved in the regulation of the inflammatory tumor microenvironment and the maintenance of cancer stem cells (CSCs) [18]. According to previous studies, p53 might function as a regulator of the NF-κB signaling pathway [19]. Several previous studies in NSCLC indicate that *Tp53* is frequently co-mutation with EGFR mutation, which is involved in survival rate of patients treated with 1st and 2nd generation tyrosine kinase inhibitors (TKI) [20]. The previous in vitro study indicates that p53 is involved in the sensitivity to EGFR-TKI [21]. The clinical relevance between survival and *TP53* indicates that *Tp53* mutation also negative regulates survival rate of the NSCLC patients with EGFR mutation and a T790M resistance mutation treated with Osimertinib [22]. Previous studies also provided evidence to support that *Tp53* mutations might be involved in primary resistance to EGFR-TKIs treatment in patients with EGFR mutations [20]. The point mutation of EGFR at position 797 were found to be involved in the resistance of Osimertinib [23]. Studying the drug-sensitive and resistant NSCLC cell lines found that p53 modulates acquired resistance to EGFR inhibitors and radiation [24]. In addition, the associations between different *Tp53* mutations and responses to targeting EGFR in NSCLC patients have been found [25]. We recently found that estrogen can increase DNMT1 expression, thereby increasing DNA methylation in the promoter of p53 and thus inhibiting p53 expression, leading to the repolarization of tumor-associated macrophages from the M1 to the M2 phenotype, which promotes cancer progression [26]. Lung cancer patients with co-mutations of EGFR and several other genes have a poor response to EGFR-TKIs [27]. However, the prognostic and predictive significance of *EGFR/Tp53* co-mutation in NSCLC patients remains controversial and needs to be clarified. In this study, we established a model of gefitinib-induced drug-resistant lung cancer in *EGFR*^L858R^*Tp53^*+/−*^ mice, which is about 35% EGFR mutation lung cancer patients, to study the effect of p53 on genomic instability and gene expression profiles.

## Results

### An in vivo lung cancer drug-resistant animal model is established by continuous gefitinib treatment

**Fig. 1:**
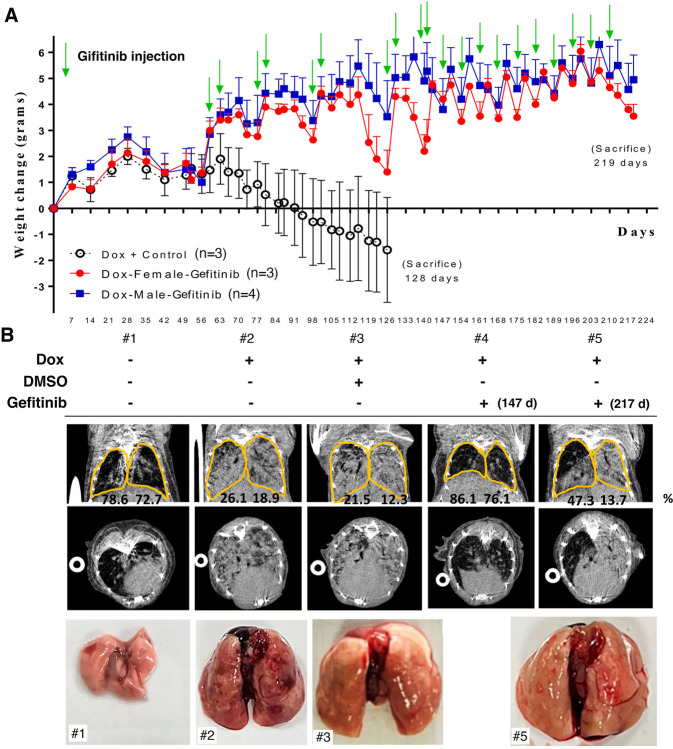
An animal model of TKI-induced drug resistance was established. Lung tumors were induced in *EGFR*^*L858R*^ mice by doxycycline (0.5 g/l in drinking water) treatment for 7 weeks, followed by treatment with 20 mg/kg gefitinib including Dox + Control (*n* = 3), Dox-Female-Gefitinib (*n* = 3) and Dox-Male-Gefitinib (*n* = 4) until drug resistance was observed, and all the mouse body weight was measured every week (**A**). In vivo tumor growth was monitored by micro-CT at different times until the establishment of drug resistance (217th day) and quantitated the air space by Sigma J software, then, the mice were sacrificed, and the lungs are shown here (**B**).

**Fig. 2:**
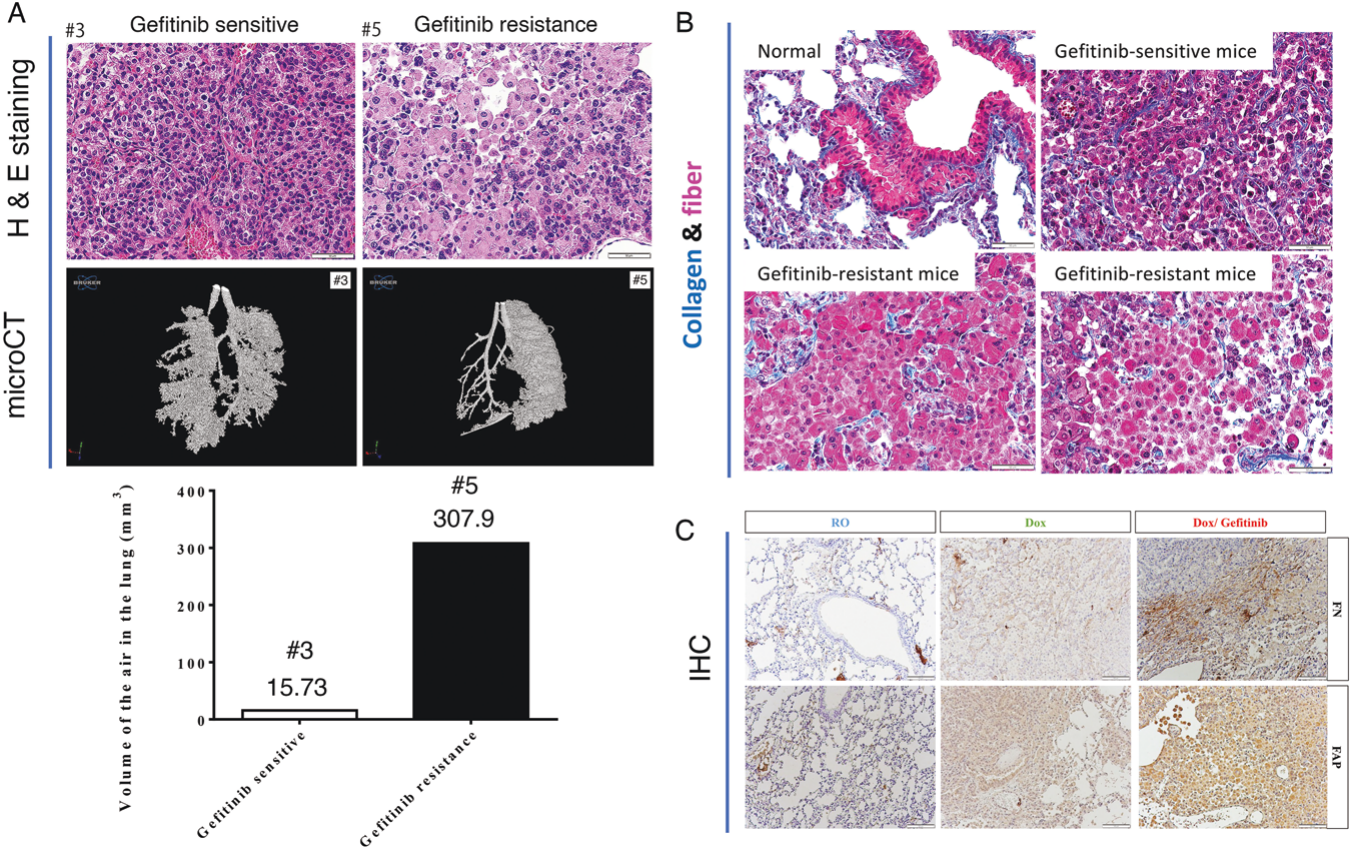
The extracellular matrix around the tumor was monitored during drug resistance acquisition. The lung pathology in gefitinib-sensitive (#3 mice) and gefitinib-resistant mice (#5 mice) was studied by H&E staining and 3D micro-CT, and then quantitated by CTAn and CTVol software (**A**). The levels of collagen and fibers in the lungs of normal mice (*n* = 3), Gefitinib-sensitive mice (*n* = 3) and Gefitinib-resistant mice (*n* = 6) were studied by Masson’s trichrome staining, blue: collagen; pink: fibers; red: cytoplasm (**B**). The levels of fibroblast markers, FN and FAP, were studied by IHC with indicated antibodies (**C**).

Most previous studies on drug resistance were performed in vitro, not in vivo. In this study, we used an in vivo mouse model of lung cancer i.e., *EGFR*^*L858R*^ mice, in which lung cancer is induced by doxycycline treatment, to establish an animal model of drug resistance by long-term treatment with 20 mg/kg gefitinib, an EGFR tyrosine kinase inhibitor (TKI). Lung tumor formation was induced by doxycycline treatment for 1.5 months in *EGFR*^*L858R*^ mice, after which continuous treatment with gefitinib was initiated [28] (Fig. 1). Two markers, body weight and the micro-CT signal of the lung, were used to monitor the treatment effects of gefitinib in mice with lung cancer (Fig. 1A, B). After cancer induction by doxycycline, the body weight of mice with lung cancer gradually decreased, and the lung micro-CT signal was dramatically increased (Fig. 1A, B). Then the drug-sensitive mice (#2 mice) were sacrificed on Day 128 (Fig. 1B). The gefitinib-treated mice were continually monitored for body weight and lung micro-CT signals. Body weight loss was reversed by weekly gefitinib treatment until Day 219 (Fig. 1A). We also used micro-CT to evaluate the tumor signal during the treatment period. At 147 days after treatment, the lung micro-CT signal was still very low, indicating a good therapeutic effect of gefitinib (Fig. 1B). However, the body weight was not restored, and the lung micro-CT signal was not abolished by gefitinib treatment on Day 219 (#5 mice), suggesting that drug resistance was induced at this time point (Fig. 1A, B). The lung size was increased in doxycycline-induced mice and in mice with gefitinib-induced drug resistant (Fig. 1B). The volume of air in the lung was about 75% air space of lung (78.6% / 72.7%) in normal mice (#1). In doxycycline-treated mice (#2 and #3), the air space of the lungs were only 26.1%/18.9% (#2) and 21.5%/12.3% (#3), respectively. After Gefitinib treatment, the air space of lung were increased (86.1%/76.1%, #4), indicating that gefitinib is effective on inhibiting cancer formation. After 217 days treatment, the air space of lung was 47.3%/13.7% (#5), suggesting that lung cancer cells were resistance to Gefitinib at this moment (Fig. 1B). Next, lung pathology was studied in gefitinib-sensitive and gefitinib-resistant mice (Fig. 2). The volume of air was 15.73/mm^3^ in gefitinib-sensitive mice (before treatment), indicating that the lung parenchyma was occupied by accumulated lung cancer cells and the cellular morphology of the lungs in drug-sensitive mice were more regular each other (Fig. 2A). In addition, the 3D micro-CT signals were 307.9/mm^3^ air volume in the drug-resistant mice (Fig. 2A, left panel)). However, the cellular morphology was very diverse each other in drug-resistant lung tumor. One type of cell was larger and contained fiber-like bodies and small nuclei (Fig. 2A). Therefore, we used Masson’s trichrome staining to evaluate the levels of collagen (blue) and fibers (pink) in normal, gefitinib-sensitive and gefitinib-resistant mice (Fig. 2B). The level of collagen was not significantly different among these mice, but the level of fibers (pink) was dramatically higher in gefitinib-resistant mice than in normal or gefitinib-sensitive mice (Fig. 2B), indicating that a type of fiber-rich cell was found in mice with drug-resistant lung cancer. In addition, the signals of fibronectin-associated protein (FAP) and fibronectin (FN), which is the markers of fibroblast, were higher in drug-resistant mice compared to drug-sensitive mice (Fig. 2C), implying that this type of cell might be a pro-fibroblast-like or fibroblast-like cell.

### Different mutational hotspot regions are found during tumorigenesis and in mice with drug-resistant lung cancer

**Fig. 3:**
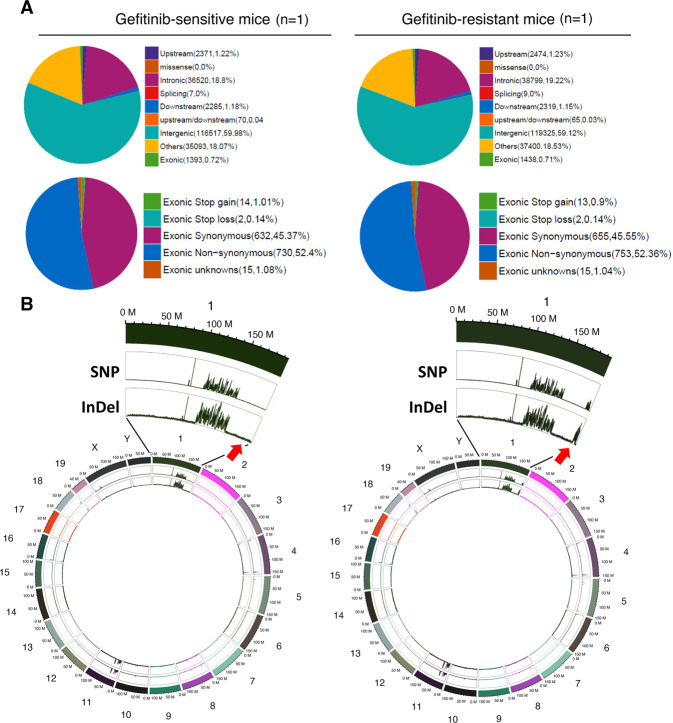
Gefitinib-induced drug resistance induces genomic instability in *EGFR*^*L858R*^-induced lung cancer mice. The percentages of sequence mutations in the various regions of the whole-genome sequence (WGS), Gefitinib-sensitive mice (*n* = 1) and Gefitinib-resistant mice (*n* = 1) are shown here (**A**). The single nucleotide polymorphism (SNP) and small insertions/deletions (InDel) profiles and the highlighted region on Chromosome 1 are shown on a Circus plot. The red arrow indicates the change during drug resistance acquisition in mice with lung cancer (**B**).

**Fig. 4:**
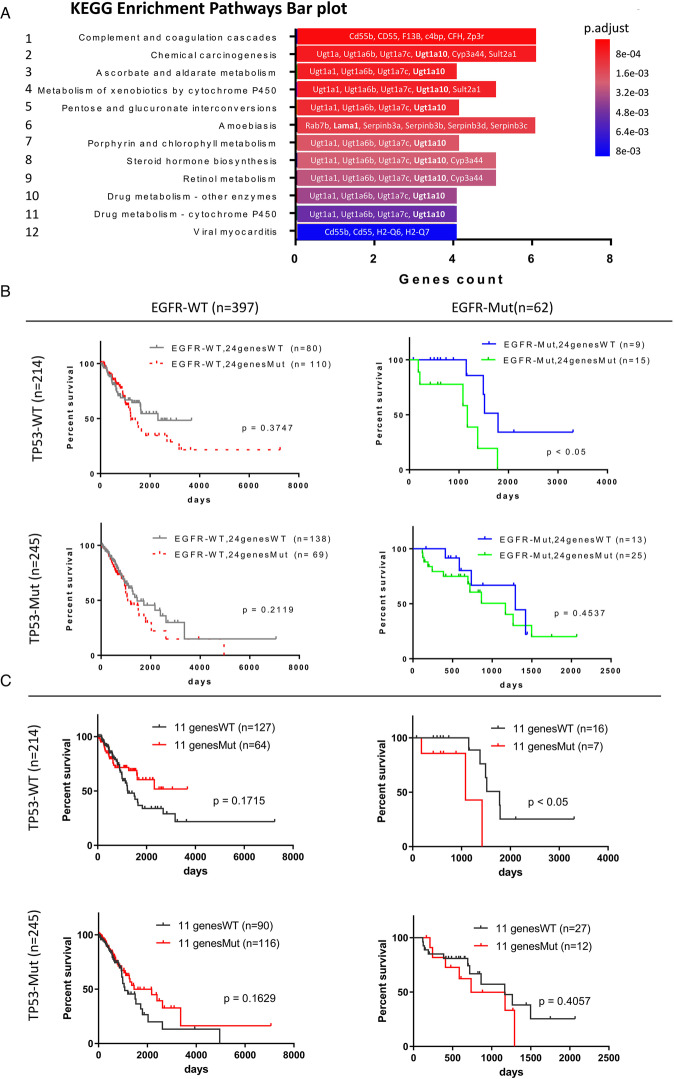
All the genes with nonsynonymous gene mutations are listed and were analyzed. Genes with nonsynonymous mutations in lung cancer drug-resistant mice were used to study the related pathways by generating KEGG pathways enrichment bar plot (**A**). All genes with nonsynonymous mutations in mice with drug-resistant lung cancer were evaluated in cohorts containing 459 patients with lung cancer including 397 EGFR-WT and 62 EGFR-Mut patients from TCGA to identify 24 genes that were also mutated in human lung cancer patients, and these genes were used to compare the survival rates in the presence or absence of p53 knockout (**B**). Eleven of the 24 genes with higher mutation frequencies in clinical lung cancer patients were used to compare the survival rates in the presence or absence of *Tp53* knockout (**C**).

Because heterogeneity is a critical factor for inducing drug resistance, genomic DNA was extracted from the lungs of gefitinib-sensitive and gefitinib-resistant mice for whole genomic sequencing (WGS) (Fig. 2A). The sequencing quality control and error rate were reasonable and are shown in the supplementary results (Supplementary Fig. 1). All the sequences were compared to the whole-genome of wild-type mice in GRCm38 genome (ftp://ftp.ensembl.org/pub/release-94/fasta/mus_musculus/dna/Mus_musculus.GRCm38.dna.primary_assembly.fa.gz). The total mutation number identified by WGS in gefitinib-resistant mice (201829 bps) was higher than that in gefitinib-sensitive mice (194258 bps) (Fig. 2A and Table 1). Analysis of the mutant distribution of mutations across the chromosomes showed that hotspot mutations in gefitinib-sensitive mice (tumorigenesis) were found on chromosome 1 (Chr.1qE2.1:4.9 cM–Chr.1qG1:63.5 cM) and chromosome 11 (Chr.11qA1:2.2 cM–Chr.11qA3.1:10.5 cM；Chr.11qA3.3:16.8 cM–Chr.11qA4:19.68 cM), indicating that this mutation profile of the gene repertoire may be related to lung tumorigenesis (Fig. 2B and Supplementary Table 1). Thirty-nine genes with nonsynonymous mutations were found in drug-sensitive mice only; these genes might be involved in lung tumorigenesis (Table 1 and Supplementary Table 1). Interestingly, a hotspot mutation in gefitinib-resistant mice was found on chromosome 1 (Chr.1qH5:89.95 cM–Chr.1qH6:97 cM) (Fig. 2C, Table 1 and Supplementary Table 2). All the gene repertoires (53 genes) containing nonsynonymous exonic mutations only in drug-resistant mice and not in drug-sensitive mice are listed in Supplementary Table 2, and more than one-third of these genes (18 genes) are located on chromosome 1, suggesting that genomic instability of chromosome 1 is induced. Next, we used KEGG pathway enrichment analysis to identify related pathways (Fig. 3A). The genes mutated under drug resistance conditions (53 genes) were involved in 12 pathways related to the complement immune system, cytochrome P450-mediated metabolism of xenobiotics, steroid hormone biosynthesis, retinol metabolism and drug metabolism-related enzymes (Fig. 4A). To study the mutation status of these genes listed in Supplementary Table 2 in human lung cancer patients, the genome sequences were obtained from TCGA. 24 genes mutated in drug-resistant mice, were also mutated in some lung cancer patients (Suppl. Table 5). Analysis of the relationships between the survival rate and these 24 genes showed no significant survival difference in EGFR-WT patients but a lower survival rate in EGFR-Mut patients with mutation of these 24 genes (*p* < 0.05) (Fig. 4B). Furthermore, if we only considered the relationships between the survival rate and the 11 genes among these 24 genes with higher mutation frequencies in TCGA clinical cohorts, poor survival in EGFR-Mut patients (*p* < 0.05) but no significant difference in survival in EGFR-WT patients was found (Fig. 4C), implying that these genes might be involved in EGFR-mutation-mediated lung cancer progression.

**Table 1:** The mutation statistics and annotation results for lung cancer formation, drug resistance and mice with *Tp53*^*+/−*^-mediated drug resistance are listed here (A). The exonic nonsynonymous mutation statistics and annotation results for lung cancer formation, drug resistance and mice with *Tp53*^*+/−*^*-*mediated drug resistance are listed here (B).

		Drug sensitive	Drug resistance	Drug sensitive	Drug resistance
		EGFR^L858R^*p53^+/+^	EGFR^L858R^*p53^+/+^	EGFR^L858R^*p53^+/−^	EGFR^L858R^*p53^+/−^
*(A) All region of mutations statistics and annotation results in EGFRL*^*858R*^*-p53*^*+/+*^ *and EGFRL*^*858R*^*-p53*^*+/−*^*mice*					
Exonic	Synonymous	633	655	806	823
	Nonsynonymous	731	753	822	783
	Stop gain	14	13	13	15
	Stop loss	2	2	2	2
	unknowns	15	15	11	11
	Intronic	36520	38799	35622	36467
	Upstream	2371	2474	2542	2544
	Downstream	2285	2319	2554	2512
	upstream/downstream	70	65	92	89
	Splicing	7	9	8	8
	Intergenic	116517	119325	112254	114578
	Others	35093	37400	38506	38738
	Total	194258	201829	193232	196570

Items	Category	Number of SNVs (Genes)			
		Drug sensitive	Drug resistance	Drug sensitive	Drug resistance
		EGFR^L858R^*p53^+/+^	EGFR^L858R^*p53^+/+^	EGFR^L858R^*p53^+/−^	EGFR^L858R^*p53^+/−^
*(B) nonsynonymous-exonic mutations data statistics and annotation results in EGFRL*^858R*^p53^*+/+*^ *and EGFRL*^858R*^p53^*+/−*^*mice*					
Only in drug-sensitive or drug-resistance	All chromosomes	74 (39)	95 (53)	74 (53)	67 (46)
	Chromosome 1	13 (7)	38 (18)	17 (6)	19 (8)
	Chromosome 11	1 (1)	3 (3)	5 (5)	4 (3)
	Other Chromosomes	60 (31)	54 (32)	52 (42)	44 (35)
In both drug sensitive and drug-resistance	All chromosomes	673 (193)	673 (193)	763 (231)	733 (231)
	Chromosome 1	325 (78)	309 (78)	315 (81)	322 (81)
	Chromosome 11	80 (27)	79 (27)	117 (54)	123 (54)
	Other Chromosomes	268 (88)	285 (88)	331 (96)	288 (96)
	Total	747 (232)	768 (246)	837 (284)	800 (277)

### *Tp53*^*+/−*^ promotes drug resistance in mice with TKI-induced drug-resistant lung cancer mice

**Fig. 5:**
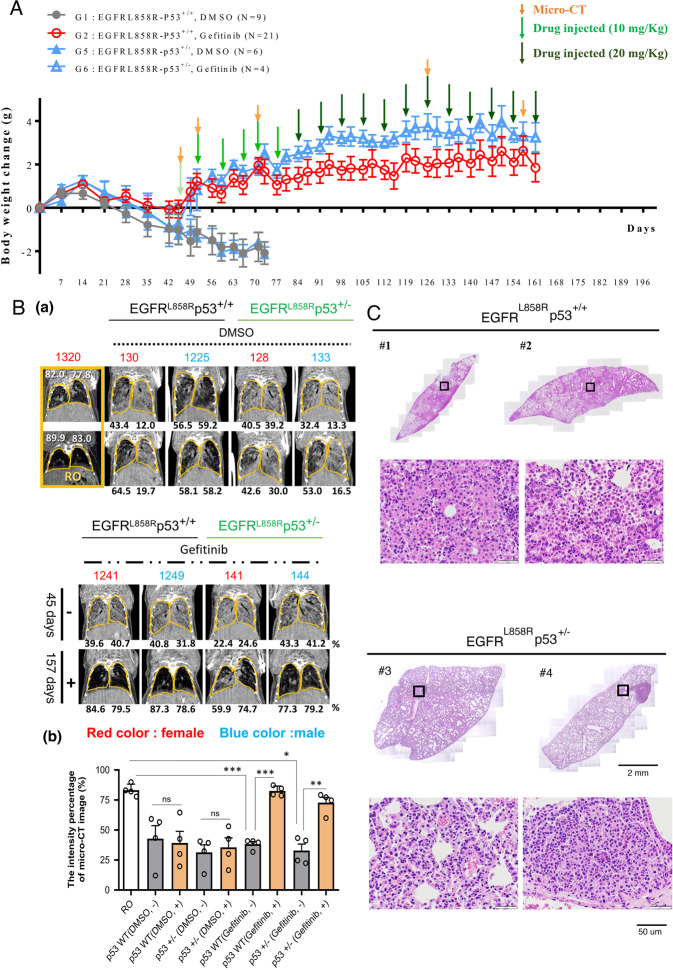
*Tp53*^+/−^ promotes drug resistance in vivo. Lung tumors were induced in *EGFR*^L858R^*Tp53^*+/+*^ and *EGFR*^L858R^*Tp53^*+/−*^ mice including *EGFR*^L858R^*Tp53^*+/+*^, DMSO (*n* = 9), *EGFR*^L858R^*Tp53^*+/+*^, Gefitinib (*n* = 21), *EGFR*^L858R^*Tp53^*+/−*^, DMSO (*n* = 6) and *EGFR*^L858R^*Tp53^*+/−*^, Gefitinib (*n* = 4) by doxycycline (0.5 g/l in drinking water) treatment for 42 days, and the mice were then treated with 10 or 20 mg/kg gefitinib (green color arrow) until the establishment of drug resistance (161th day). The body weight of all the mice was measured every week (**A**), and the tumor growth was studied by micro-CT in the different stages (orange color arrow) (**B**, **a**) The signal (block color) of air space in the lungs was determined by Image J, and then the statistical analysis was performed by *t* test; *p < 0.05, **p < 0.01, ***p < 0.005, ns: non-significance (**B**, **b**). The pathology of mice with drug-resistant lung cancer mice with or without *Tp53*^*+/−*^ was studied by H&E staining (**C**).

Loss of p53 will induce genomic instability in the different cell types. What is the effect of *TP53* mutation on genomic instability during drug resistance acquisition? In this study, we have constructed gefitinib-induced drug-resistant lung cancer mice. Herein we used *EGFR*^L858R^*Tp53^*+/+*^ and *EGFR*^L858R^*Tp53^*+/−*^ mice to study the effect of the *Tp53*^*+/−*^ genotype on genomic integrity (Fig. 5). To induce drug resistance quickly, we did some treatment modification. We used lower dose, 10 mg/kg, of gefitinib to treat mice at early period, and then used higher dose, 20 mg/kg, at a late period to induce drug resistance (Fig. 5). The body weight and lung micro-CT signal were used to monitor the inhibitory effect of gefitinib on cancer formation (Fig. 5A, B). The body weight of mice with doxycycline-induced lung cancer was decreased, but the body weight was increased after gefitinib treatment (Fig. 5A). After continuous treatment for 161 days, the body weight did not increase further, implying that drug resistance was induced at this time point (Fig. 5A). The lung micro-CT signal was higher in mice with doxycycline-induced lung tumor but the signal was abolished after gefitinib treatment until Day 161 (Fig. 5B). The findings indicated that the micro-CT signal in *EGFR*^L858R^*Tp53^*+/−*^ mice was higher than that in *EGFR*^L858R^*Tp53^*+/+*^ mice, suggesting that partial loss of p53 (*Tp53*^*+/−*^) was likely to induce drug resistance (Fig. 5B). Next, the drug-resistant mice were sacrificed to study the pathology by H&E staining (Fig. 5C). The results indicated that cancer cells were accumulated in *EGFR*^L858R^*Tp53^*+/−*^ mice and *EGFR*^L858R^*Tp53^*+/+*^ mice (Fig. 5C, (a)). Consistent with the results in Fig. 1, the myofibroblast-like cells were found in drug-resistant tumors in *EGFR*^L858R^*Tp53^*+/+*^ and *EGFR*^L858R^*Tp53^*+/−*^ mice, more intense eosin staining, a higher N/C ratio and irregular in the nuclear morphology were found in the lungs from *EGFR*^L858R^*Tp53^*+/−*^ mice than in those from *EGFR*^L858R^*Tp53^*+/+*^ mice, implying that partial loss of p53 led to the induction of drug resistance during cancer therapy (Fig. 5C).

### *Tp53*^*+/−*^-genotype-mediated hotspot mutation on chromosome 11 in mice with drug-resistant lung cancer

**Fig. 6:**
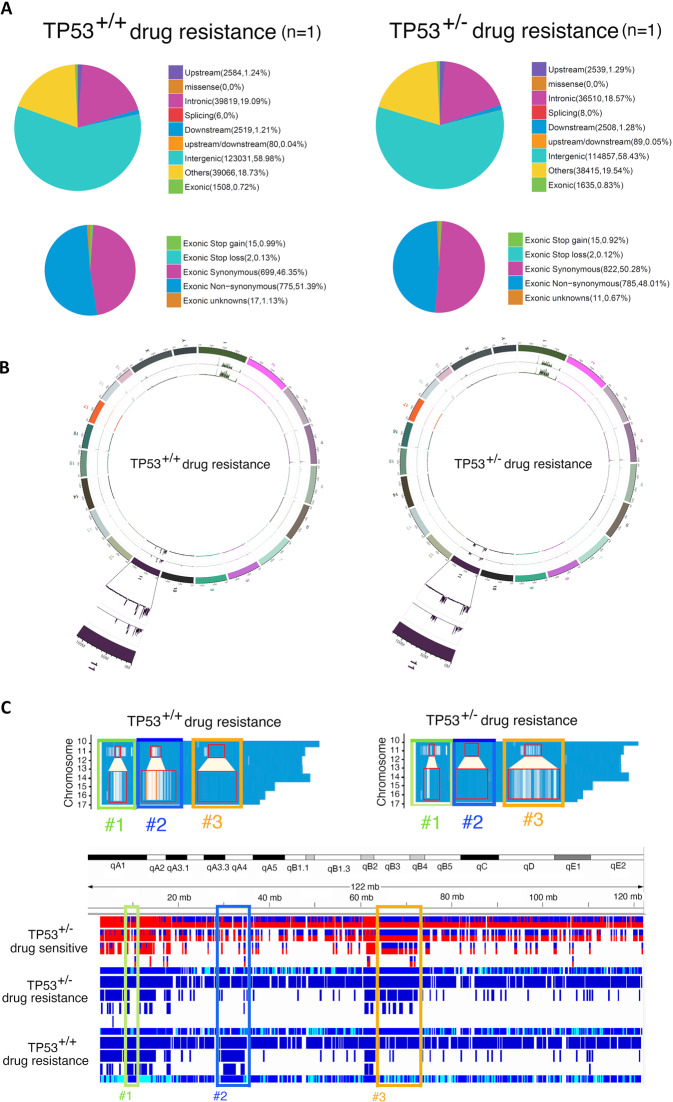
The effect of *Tp53*^*+/−*^ on genomic mutations in drug-resistant mice. Total genomic RNA was isolated from lung tissues of drug-resistant mice (*EGFR*^*L858R*^) with or without the *Tp53*^*+/−*^ genotype, *EGFR*^L858R^*Tp53^*+/+*^ (*n* = 1) and *EGFR*^L858R^*Tp53^*+/−*^ (*n* = 1), for whole-genome sequencing (WGS). The percentages of sequence mutations in the various regions of the whole-genome sequence (WGS) are shown here (**A**). The SNP and InDel profiles in drug-resistant mice with or without the *TP53*^*+/−*^ mice were used to study the related pathways by Circus plot. The Chromosome 11 was highlighted (**B**). Genes with nonsynonymous mutations in the Chromosome 11 of drug-resistant mice with or without *Tp53*^*+/−*^ were shown here in detail (**C**).

WGS data from *EGFR*^L858R^*Tp53^*+/−*^ mice and *EGFR*^L858R^*Tp53^*+/+*^ mice were prepared to study the effect of p53 mutation on genomic instability (Fig. 6). The quality control and error rate of the exome sequencing data were reasonable (Supplementary Fig. 1). The number of mutations in exonic regions in *EGFR*^L858R^*Tp53^*+/−*^ mice (1635, 0.83%) was higher than that in *EGFR*^L858R^*Tp53^*+/+*^ mice (1508, 0.72%) (Fig. 6A and Table 1). The numbers of mutations (synonymous / Nonsynonymous) in exonic regions in *EGFR*^L858R^*TP53^*+/−*^ mice (823/783) were higher than those in *EGFR*^L858R^*Tp53^*+/+*^ mice (655/753) (Fig. 6A and Table 1A). The total number of genes with nonsynonymous mutations was increased in *Tp53*^*+/−*^ drug-resistant mice (*Tp53*^*+/+*^/*Tp53*^*+/−*^ = 193/231) (Table 1B). The hotspot mutations in both the *EGFR*^L858R^*Tp53^*+/−*^ drug-sensitive and resistant mice were primarily found on chromosome 1 (*Tp53*^*+/+*^/*Tp53*^*+/−*^ = 78/81) and chromosome 11 (*Tp53*^*+/+*^/*Tp53*^*+/−*^ = 27/54) (Fig. 6B, Fig. 6C and Table 1B). In addition, 53 genes with nonsynonymous mutations were found in *Tp53*^*+/−*^ mice only, but another 46 genes were found in the drug-resistant *Tp53*^*+/−*^ mice only (Table 1B, Supplementary Table 3 and Supplementary Table 4). Interestingly, partial loss of p53 (*Tp53*^*+/−*^) facilitated the mutation on chromosome 11 (11qB3:40.4 cM-11qB4:44.4 cM), but other regions on chromosome 11 (Chr.11qA1:6.1 cM–Chr.11qA1:6.3 cM；Chr.11qA3.2:16.8 cM–Chr.11qA5:19.9 cM), which were mutated in *EGFR*^L858R^*Tp53^*+/+*^ drug-resistant mice, were not mutated in EGFR^L858R^*Tp53^+/−^ drug-resistant mice (Fig. 6C, #2). In summary, high amount of mutations were found in qB2-qB4 region of Chromosome 11 (#3) in *Tp53*^*+/−*^ drug-sensitive and resistant mice but not in qA region (#1 and #2). However, lower mutations were found in qB2-qB4 region of Chromosome 11 (#3) in *Tp53*^*+/+*^ drug-resistant mice but higher mutations in the qA region (#1 and #2).

### Drug resistance-related genes are induced in drug-resistant lung cancer mice

**Fig. 7:**
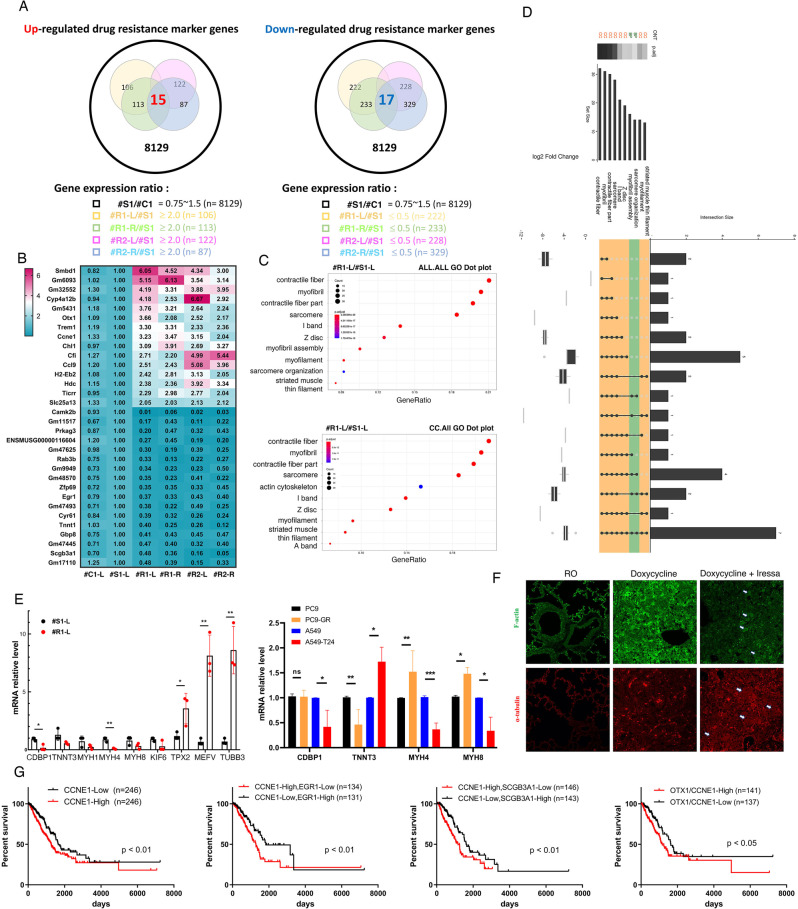
Genes were regulated during drug resistance acquisition but not during tumorigenesis in mice with TKI-induced drug resistance. RNA samples isolated from normal (*n* = 1; #C1), drug-sensitive (*n* = 1; #S1) and drug-resistant (n = 2; #R1-L, #R1-R, #R2-L and #R2-R) mice were used to study the gene expression by RNA-seq. Up- and downregulated genes in drug-resistant mice were studied by identifying the overlapping genes between drug-sensitive and drug-resistant mice (**A**). Gene expression during drug resistance acquisition was studied by RNA-seq and is shown here on a heatmap (**B**). The significance of the GO terms regulated by drug resistance was studied by generating ALL and CC GO dot plots (**C**) and a GO Upset plot (**D**). All genes showing change only in mice with drug resistance by RNA-seq were used for validation by qPCR with more samples from drug-resistant mice and various drug-resistant cell lines, PC9, Gefitinib-induced drug-resistant PC9 (PC9-GR), A549 and Taxol-induced drug-resistant A549 (A549-T24). The statistical assay was performed by *t* test after three independent experiments; *p < 0.05, **p < 0.01, ***p < 0.005, ns: non-significance (**E**). The signals of F-actin (green) and α-tubulin (red) in the lungs of drug-sensitive and drug-resistant mice with lung cancer were studied by IF with anti-actin and anti-tubulin antibodies (**F**). The relationships between the survival rates in lung cancer cohorts from TCGA and the levels of several genes (*CCNE1, CCNE1/EGR1, CCNE1/SCGB3A1 and OTX1/CCNE1*) in the lungs of drug-resistant mice (**G**).

As shown in Fig. 1 and Fig. 2, we used 3 drug-sensitive and 7 drug-resistant mice to address the difference of pathology between drug-sensitive and drug-resistant lung tumors. The gene expression profile and related pathways were then explored. RNA was isolated from drug-sensitive and drug-resistant lung cancer mice, *EGFR*^L858R^*Tp53^*+/+*^, to study the systemic gene expression profile by RNA-seq (Fig. 7). The quality of these samples was reliable and is shown in Supplementary Fig. 2. We used these RNA-Seq data to analyze global gene expression (Fig. 7). Upregulation of 15 genes and downregulation of 17 genes were found in the 4 drug-resistant samples (#R1-L, #R1-R, #R2-L and #R2-R) but not in the drug-sensitive sample (#S1) (Fig. 7A, Fig. 7B and Supplementary Table 6). Next, we used a GO dot plot (Fig. 7C) and a GO Upset plot (Fig. 7D) to study the signaling pathways of drug resistance-related genes (#R1-L/#S1). Many genes involved in drug-resistant lung cancer were related to fibers and the cytoskeleton, such as contractile fibers, myofibrils, sarcomeres and myofilaments, for which the p values were significant (Fig. 7C, D). Finally, samples from mice (#R1-L and #S1) and NSCLC cell lines (PC9 and A549) and their drug-resistant cell lines, gefitinib-resistant PC9 (PC9-GR) and Taxol-resistant A549 (A549-T24) were used to validate the results of RNA-Seq related to cytoskeleton-related genes (Fig. 7E). The levels of actin-related genes were decreased, and those of microtubule-related genes were increased in drug-resistant mice and cell lines (Fig. 7E). Because many cytoskeletal genes were altered in drug-resistant mice, the cytoskeletal remodeling might occur during cancer therapy. The results indicated that the signal of F-actin in the tumor region of the drug-resistant mice was decreased in most of the drug-resistant cells (Fig. 7F, up panel). On the other hand, the signal of α-tubulin was increased in most of the drug-resistant cells in the tumor region of drug-resistant mice (Fig. 7F, low panel), suggesting that cytoskeletal remodeling occurred during drug resistance. Finally, the relationships between the survival rate and expression of drug resistance-related genes were studied (Fig. 7G). The data indicated that the CCNE1-high, CCNE1-high/EGR1-low, CCNE1-high/SCGB3A1-low and OTX1-high/CCNE1-high phenotypes, which might be involved in lung cancer malignancy, are related to poor prognosis in lung cancer.

### *Tp53*^*+/−*^-mediated gene expression profile in mice with drug-resistant lung cancer

**Fig. 8:**
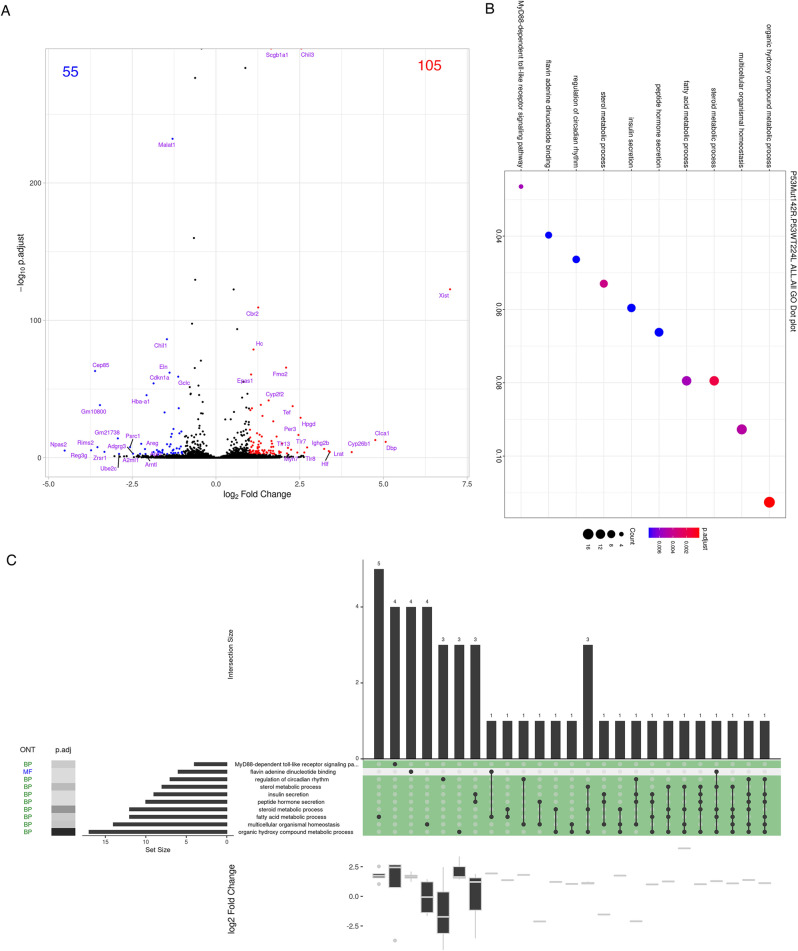
Gene expression during drug resistance acquisition was regulated in a *Tp53*^*+/+*^ manner. mRNA samples isolated from lung tissues of drug-resistant mice with (*n* = 1) or without *Tp53* knockout (*n* = 1) were used to study the gene expression by RNA-seq. The gene changes in drug-resistant mice with or without *Tp53* knockout were studied by generating a DEG plot/volcano plot (**A**). The p53-mediated pathways during drug resistance acquisition were analyzed by generating a GO dot plot (**B**). Enriched intersecting pathways were analyzed by generating by a GO UpSet plot (**C**).

Here, we have studied the global gene expression profile in *Tp53*-WT drug-resistant mice (Fig. 7). Because many cancer patients have *TP53*^*+/−*^, we also used drug-resistant mice with *Tp53* haploinsufficiency (*EGFR*^L858R^*Tp53^*+/−*^) to study the effect of p53 mutation on global gene expression during drug resistance acquisition (Fig. 8). A total of 105 genes were upregulated, and 55 genes were negatively regulated under the loss of p53 in drug-resistant mice (Fig. 8A). Next, these genes were analyzed with a GO-dot plot (Fig. 8B) and a GO Upset plot (Fig. 8C). Several pathways, including the MyD88-dependent Toll-like receptor signaling pathway, lipogenesis-related gene expression repertoires and regulation of circadian rhythm were differentially regulated in *TP53*-mutated drug-resistant mice (Fig. 8B, C). Loss of p53 increased the expression of most genes related to the MyD88-dependent Toll-like receptor signaling pathway, which is related to the NF-κB pathway (Fig. 8B, C). In addition, most of the lipogenesis-related pathways, such as fatty acid metabolic process, steroid metabolic process, sterol metabolic process and insulin secretion were upregulated in p53-mutant mice with drug-resistant lung cancer (Fig. 8B, C). Interestingly, several genes related to the regulation of circadian rhythm were significantly downregulated under the loss of p53, suggesting that p53 might be involved in controlling circadian rhythm (Fig. 8B, C).

## Discussion

Herein, we used an intact in vivo model of drug-resistant lung cancer to study genomic mutations and global gene expression profiles. During gefitinib-induced drug resistance in mice, cytoskeletal remodeling-related genes were regulated to affect cancer progression. Hotspot mutations were induced on chromosome 1 and chromosome 11 in a *Tp53*^*+/+*^ and *Tp53*^*+/−*^ manner respectively, during drug resistance acquisition.

Tumor heterogeneity develops during cancer progression from initiation to malignant transformation [29]. Furthermore, therapeutic periods also promote tumor heterogeneity through an increase in genomic instability, thereby inducing drug resistance [30]. Tumor heterogeneity can be classified as intra-tumoral (within a tumor) or intertumoral (between tumors) [31]. Most of the regulatory factors can affect both intra- and intertumoral heterogeneity. In this study, the TKI gefitinib was used for long-term treatment of mice with *EGFR*^*L858R*^- and *EGFR*^L858R^*Tp53^*+/−*^*-*induced lung cancer to induce drug resistance and then study the mutation burden and gene expression profile. Although the somatic mutation variants (e.g., SNPs) in the different mice could contribute to differences in the whole-genome sequence among the mice, the hotspot mutation repertoires on chromosome 1 and chromosome 11 were dramatically enhanced when compared with the SNPs. Therefore, we think that the major contribution to the hotspot mutations were from the gefitinib treatment. In addition, all the mutated genes in mice with *EGFR*^*L858R*^-induced lung cancer and gefitinib-induced drug resistance were evaluated in the genomic sequences of lung cancer patients from TCGA. Approximately 25% of these genes were also mutated in the lung cancer cohorts. Species specificity may lead to the differences in the mutation profiles between mice and humans [32]. In addition, the cancer stages and the treatment histories of lung cancer patients in TCGA were different, which could also result in different mutation profiles. Finally, when we studied the survival rates of patients with lung cancer with wild-type and mutant genotypes, the patients with EGFR mutation had significantly poorer prognosis than those with wild-type EGFR, indicating an interaction between the EGFR signaling pathway and drug resistance-induced gene mutation. One possibility is that hotspot mutations are likely to be induced in patients with EGFR mutations. The other possibility is that the EGFR signaling pathway can regulate mutant gene expression, subsequently leading to drug resistance. Although monoclonal antibodies (mAbs) and TKIs have been clinically used to treat lung cancer patients, drug resistance and recurrence still occur after long-term treatment [33, 34]. According to previous studies, gefitinib-induced drug resistance may occur through T790M mutation of *EGFR*, amplification of MET or HER2, or overexpression of AXL or the hepatocyte growth factor (HGF) [35, 36]. In addition, mutations in the RAS and BRAF genes have been found to mediate TKI-induced drug resistance during lung cancer therapy [37]. In this study, we cannot find T790M mutation in the drug resistance mice. It might be due to that the transgenic mice, *EGFR*^*L858R*^, which did not mutate the endogenous EGFR gene but constructed another transgene, *EGFR*^*L858R*^, into the chromosome to induce lung cancer. Another possibility is that T790M mutation in human lung patients is strain specificity. In this study, several mutation genes, Ugt1A, CD55, Cyp3a44, Sult2a1, CFH, Zp3r, Rab7b, Lama1, Serpinb3, were found on chromosome 1 in *EGFR*^*L858R*^-induced drug-resistant mice, which might be related to drug resistance. UDP Glucuronosyltransferase Family 1 Member A Complex Locus (UGT1A) polymorphisms associated with worse outcome in colorectal cancer patients treated with irinotecan-based chemotherapy [38], indicated that UGT1A genetic variants might potentially be developed as a biomarker of drug resistance during cancer therapy. Overexpression of CD55 in the tumor microenvironment protects cancer cells from complement-mediated attack [39]. SULT2A1 genetic variants does not impact on actual DHEA/DHEAS ration and HCC [40, 41]. Human completement factor H (CFH) is an important complement control protein, which is a novel diagnostic marker for lung adenocarcinoma [42]. Rab7 belongs to Ras oncogene family, and its dysfunction is associated with cancer malignancy [43]. Whether these genes are involved in these drug resistance-related pathways needs to be addressed in the future.

p53, as a tumor suppressor, plays a critical role in maintaining genomic integrity by regulating cell cycle checkpoints, DNA repair and apoptosis [44]. *Tp53* mutation can be divided into two types, gain-of-function and loss-of-function, both of which are involved in genomic instability under the stressful environmental conditions, such as those imposed by toxin stress or cancer therapy [45, 46]. Various residues located in different regions of p53 can be mutated during cancer progression and the therapeutic period. Many residues located in the DNA binding domain of p53 can be mutated to abolish its DNA binding activity, thereby causing p53 to lose its function as a transcriptional factor to regulate its target genes, which are involved in controlling cell cycle checkpoint-, DNA damage repair- and apoptosis-related genes [47, 48]. In this study, we used deletion of *Tp53* exon 5, whose encoded residues are located within the DNA binding domain, to abolish p53 DNA binding activity in mice to study its effect on mutation burden during the acquisition of TKI-induced drug resistance. We found that loss of *TP53* induced hotspot mutations on chromosome 11 near the *Tp53* locus in drug sensitive and resistant mice, indicating *Tp53*^+/−^-mediated hotspot mutations in this region, qB arm of chr.11, are not related to drug resistance. This phenomenon corresponds to *Tp53*^*+/−*^ on chromosome 17 in human cancer patients [49]. However, several genes on chromosome 11 that were mutated in *Tp53*^*+/+*^ drug-resistant mice were protected in *Tp53*^*+/−*^ mice, implying that TP53 mutation may reduce the mutation pressure for this region, the qA arm of chr.11. This means that the gene mutation in the q-arm of chr.11 is distinguishable between the mice with or without *Tp53* haploinsufficiency (*Tp53*^*+/−*^). However, the detailed molecular mechanism of which p53 mutation can protect some genes from mutation needs to be addressed in the future.

The cytoskeleton plays an important role in controlling the cell shape, which is involved in cell motility and intracellular transport [50, 51]. In addition, the cytoskeleton serves as a scaffold for signaling pathways to control signal transduction [52, 53]. In this study, obvious cytoskeletal remodeling was found in the drug-resistant cells to affect the cell shape and drug sensitivity. Many studies on cytoskeletal remodeling during cancer progression have been conducted [54, 55]. In this study, the amount of actin filaments was significantly decreased but that of microtubules was increased in drug-resistant cells compared to drug-sensitive cells, implying that cytoskeletal remodeling was triggered during drug resistance acquisition. In addition, by H&E staining and Masson staining, these fiber-like features, and the signals of FAP and FN were identified in these large cells, which were similar to myofibroblasts. Herein, we only used the fiber-rich property to identify these specific large cells, we called this type of cell a myofibroblast-like cells, and these cells were specifically identified among drug-resistant cells, implying that this type of cell is involved in drug resistance. Because myofibroblasts can be derived from fibroblasts and cancer cells [56, 57], the cell type that can be changed to become this myofibroblast-like cell needs to be identified in the future. Interestingly, several crucial pathways were differentially regulated in drug-resistant mice in the absence of p53, suggesting that these pathways are regulated by p53 during drug resistance acquisition. First, p53 can activate the MyD88-dependent Toll-like receptor signaling pathway, which is thought to promote inflammation and resistance to apoptosis and to induce a stem-like phenotype during the progression of cancers such as ovarian cancer [58, 59]. Second, loss of p53 can increase lipogenesis-related genes expression, which may be related to drug resistance. Cancer-associated adipocytes are involved in cancer progression and can remodel the extracellular matrix [60, 61]. The importance of p53-mediated pathways in drug resistance needs to be addressed in the future.

## Material and methods

### Cell culture

Human lung adenocarcinoma epithelial cell lines A549 and PC9 were purchased from ATCC. Taxol-induced A549 drug-resistant cell (A549-T24) was induced by Taxol treatment for three months in our lab. Gefitinib-induced PC9 drug-resistant cell (PC9-GR) was kindly provided by Professor WC Sunny Su from the medical center of National Cheng Kung University. All the lung cancer cell lines are cultured with RPMI 1640 medium (Invitrogen) containing 10% fetal bovine serum (FBS), 100 μg/ml streptomycin and 100 U/ml penicillin G sodium. All cells are incubated at 37 °C with 5% CO_2_. For transfecting plasmid, Polyjet (SignaGen) is used according to the manufacturer’s instructions.

### Animal cares and animal models

The experiments related to animals were approved by the Institutional Animal Care and Use Committee (IACUC:108005) at National Cheng Kung University (NCKU). These transgenic mice were generated in National Laboratory Animal Center (NLAC, Taiwan, Tainan). After breeding, two-month-old transgenic mice were used to study lung cancer development. Caging was provided suitable space and accommodates appropriate population densities that allowed animals’ sufficient freedom of movement. The enough food for transgenic mice normal growth and maintenance of normal body weight were provided. These transgenic mice were accessed to fresh and uncontaminated drinking water. Transgenic mice were also observed and cared at least for two to three times per week. All methods involved animal studies were performed in accordance with the relevant guidelines and regulations. The conditional lung cancer mice used in this study, B6;*CBA-Tg(tetO-EGFR*^*L858R*^)56Hev*B6.Cg-Tg(Scgbla-rtTA)1Jaw/J and B6;*CBA-Tg(tetO-EGFR*^*L858R*^)56Hev*B6.Cg-Tg(Scgbla-rtTA)1Jaw/J * C57BL/6-p53^*+/−*^, which can be induced by doxycycline treatment (28).

### Micro-CT

Micro-CT images of the lungs were obtained using a SkyScan 1276 (at Bruker Biospin, Billerica, MA). Mice were anesthetized with isoflurane. Scans were performed with the following parameters: source voltage and current (60 kV and 200 μA), imaging pixel size was 34.9999451 μm with 0.5 μm filter. The distance of object to source was set to 79.138 mm. Total scan time was approximately 20 min for the lung. Image processing were performed with DataViewer (Bruker) software and then rendered into 3D images in CTAn and CTVol software (Bruker).

### RNA-Seq

Total RNA was extracted and purified by a Quick-RNA MiniPrep Kit (Zymo Research, Irvine, CA, USA). For RNA Sequencing analysis, 3 μg total RNA were qualified and sequenced by Biotools Biotech Co. Ltd. (Taipei City, Taiwan). The detailed methods were shown in the supplementary material. All the primers used in this study were listed in Supplementary Table 7.

### Whole-genome sequencing

The drug-sensitive and drug-resistant mice with *EGFR*^*L858R*^ or *EGFR*^L858R^*TP53^*+/−*^ were sequenced for their whole genomes. Genomic DNA materials were extracted by using QIAamp DNA Mini kit (QIAGEN, Germantown, MD, USA.) according to the manufacturer’s protocol (Supplementary material and methods).

### Statistical analysis

All samples were used for statistical analysis. The difference between two groups was analyzed by Student’s *t* test. The *P*-value, which is smaller than 0.05, was considered as statistically significant. s.e.m. is used to calculate and plot error bars from raw data.

## Supplementary information

Supplementary Figure 1

Supplementary Figure 2

Supplementary Table 1

Supplementary Table 2

Supplementary Table 3

Supplementary Table 4

Supplementary Table 5

Supplementary Table 6

Supplementary Table 7

Supplementary material and methods

## Data Availability

All data available in the main text or the supplementary materials.
