# Supplementary material for: Tp53 haploinsufficiency is involved in hotspot mutations and cytoskeletal remodeling in gefitinib-induced drug-resistant EGFRL858R-lung cancer mice
Source: Cell Death Discov. 2023 Mar 14;9:96. doi: 10.1038/s41420-023-01393-2 (PMC10015023; doi:10.1038/s41420-023-01393-2)
Supplement: Supplementary file 3 — Supplementary Table 1 [file 41420_2023_1393_MOESM3_ESM.docx]

Supplementary Table 1. The tumorigenesis gene list in EGFR^L858R^-p53^+/+^ mice

| Ensembl Gene ID | Gene Name | Gene Full Name | CHROM（mice) | CHROM（human) |
| --- | --- | --- | --- | --- |
| ENSMUSG00000006546 | Cryba2 | crystallin, beta A2(Cryba2) | 1 | 2 |
| ENSMUSG00000026432 | Avpr1b | arginine vasopressin receptor 1B(Avpr1b) | 1 | 1 |
| ENSMUSG00000044340 | Phlpp1 | PH domain and leucine rich repeat protein phosphatase 1(Phlpp1) | 1 | 18 |
| ENSMUSG00000026222 | Sp100 | nuclear antigen Sp100(Sp100) | 1 | 2 |
| ENSMUSG00000006014 | Prg4 | proteoglycan 4 (megakaryocyte stimulating factor, articular superficial zone protein)(Prg4) | 1 | 1 |
| ENSMUSG00000006299 | Aamp | angio-associated migratory protein(Aamp) | 1 | 2 |
| ENSMUSG00000033898 | Cfhr2 | complement factor H-related 2(Cfhr2) | 1 | 1 |
| ENSMUSG00000035033 | Tbr1 | T-box brain transcription factor 1(Tbr1) | 2 | 2 |
| ENSMUSG00000027386 | Fbln7 | fibulin 7(Fbln7) | 2 | 2 |
| ENSMUSG00000079277 | Hoxd3 | homeobox D3(Hoxd3) | 2 | 2 |
| ENSMUSG00000084897 | [Gm14226](http://www.informatics.jax.org/marker/MGI:3649244) | predicted gene 14226([Gm14226](http://www.informatics.jax.org/marker/MGI:3649244)) | 2 | - |
| ENSMUSG00000054417 | Cyp3a44 | cytochrome P450, family 3, subfamily a, polypeptide 44(Cyp3a44) | 5 | 7 |
| ENSMUSG00000067700 | Gm5862 | predicted gene 5862(Gm5862) | 5 | - |
| ENSMUSG00000037999 | Arap2 | ArfGAP with RhoGAP domain, ankyrin repeat and PH domain 2(Arap2) | 5 | 4 |
| ENSMUSG00000096878 | Gm21083 | predicted gene 21083(Gm21083) | 5 | - |
| ENSMUSG00000030867 | Plk1 | polo like kinase 1(Plk1) | 7 | 16 |
| ENSMUSG00000078817 | Nlrp12 | NLR family, pyrin domain containing 12(Nlrp12) | 7 | 19 |
| ENSMUSG00000064194 | Zfp936 | zinc finger protein 936(Zfp936) | 7 | 7,19 |
| ENSMUSG00000059146 | Ntrk3 | neurotrophic tyrosine kinase, receptor, type 3(Ntrk3) | 7 | 15 |
| ENSMUSG00000030911 | Zp2 | zona pellucida glycoprotein 2(Zp2) | 7 | 16 |
| ENSMUSG00000043354 | Or51g2 | olfactory receptor family 51 subfamily G member 2(Or51g2) | 7 | 11 |
| ENSMUSG00000096679 | Or5p73 | olfactory receptor family 5 subfamily P member 73(Or5p73) | 7 | 11 |
| ENSMUSG00000041949 | Tango6 | transport and golgi organization 6(Tango6) | 8 | 16 |
| ENSMUSG00000061958 | Defa38 | defensin, alpha, 38(Defa38) | 8 | 8 |
| ENSMUSG00000019731 | Slc35e1 | solute carrier family 35, member E1(Slc35e1) | 8 | 19 |
| ENSMUSG00000032803 | Cdv3 | carnitine deficiency-associated gene expressed in ventricle 3(Cdv3) | 9 | 3 |
| ENSMUSG00000075046 | Duxf3 | double homeobox family member 3(Duxf3) | 10 | - |
| ENSMUSG00000071866 | [Ppia](http://www.informatics.jax.org/marker/MGI:97749) | peptidylprolyl isomerase A(Ppia) | 11 | 7 |
| ENSMUSG00000042724 | Map3k9 | mitogen-activated protein kinase kinase kinase 9(Map3k9) | 12 | 14 |
| ENSMUSG00000056223 | Spata31 | spermatogenesis associated 31(Spata31) | 13 | 9 |
| ENSMUSG00000095909 | Zfp997 | zinc finger protein 997(Zfp997) | 13 | 4, 19 |
| ENSMUSG00000021763 | Cspg4b | chondroitin sulfate proteoglycan 4B(Cspg4b) | 13 | 5 |
| ENSMUSG00000074832 | Zfp998 | zinc finger protein 998(Zfp998) | 13 | 7, 19 |
| ENSMUSG00000021606 | Ndufs6 | NADH:ubiquinone oxidoreductase core subunit S6(Ndufs6) | 13 | 5 |
| ENSMUSG00000022194 | Pabpn1 | poly(A) binding protein, nuclear 1(Pabpn1) | 14 | 14 |
| ENSMUSG00000036298 | Slc2a13 | solute carrier family 2 (facilitated glucose transporter), member 13(Slc2a13) | 15 | 12 |
| ENSMUSG00000060224 | Pyroxd2 | pyridine nucleotide-disulphide oxidoreductase domain 2(Pyroxd2) | 19 | 10 |
| ENSMUSG00000072049 | Vmn2r121 | vomeronasal 2, receptor 121(Vmn2r121) | X | - |
| [ENSMUSG00000095637](http://asia.ensembl.org/Mus_musculus/Gene/Idhistory?g=ENSMUSG00000095637) | Gm21608 | predicted gene 21608 (Gm21608) | X | - |

- These genes mutation repertoire are only found in gefitinib-sensitive EGFR^L858R^-p53^+/+^ lung cancer mice.
