# Supplementary material for: Tp53 haploinsufficiency is involved in hotspot mutations and cytoskeletal remodeling in gefitinib-induced drug-resistant EGFRL858R-lung cancer mice
Source: Cell Death Discov. 2023 Mar 14;9:96. doi: 10.1038/s41420-023-01393-2 (PMC10015023; doi:10.1038/s41420-023-01393-2)
Supplement: Supplementary file 4 — Supplementary Table 2 [file 41420_2023_1393_MOESM4_ESM.docx]

**Supplementary Table 2. The drug-resistance gene list in EGFR^L858R^-p53^+/+^ mice**

| **Ensembl Gene ID** | | **Gene Name** | **Gene Full Name** | **CHROM**（mice) | **CHROM**（Human) |
| --- | --- | --- | --- | --- | --- |
| ENSMUSG00000026604 | Ptpn14 | | protein tyrosine phosphatase, non-receptor type 14(Ptpn14) | 1 | 14 |
| ENSMUSG00000026630 | Batf3 | | basic leucine zipper transcription factor, ATF-like 3(Batf3) | 1 | 1 |
| ENSMUSG00000037375 | Hhat * | | hedgehog acyltransferase(Hhat) | 1 | 1 |
| ENSMUSG00000026609 | Ush2a | | usherin(Ush2a) | 1 | 1 |
| ENSMUSG00000057072 | Spata45 | | spermatogenesis associated 45(Spata45) | 1 | 1 |
| ENSMUSG00000062510 | Nsl1 | | NSL1, MIS12 kinetochore complex component(Nsl1) | 1 | 1 |
| ENSMUSG00000026150 | Mff | | mitochondrial fission factor(Mff) | 1 | 2 |
| ENSMUSG00000066595 | Flvcr1 | | feline leukemia virus subgroup C cellular receptor 1(Flvcr1) | 1 | 1 |
| ENSMUSG00000073631 | [Gm10553](http://www.informatics.jax.org/marker/MGI:3642178) | | predicted gene 10553 (Gm10553) | 1 | - |
| ENSMUSG00000058248 | Kcnh1 * | | potassium voltage-gated channel, subfamily H (eag-related), member 1(Kcnh1) | 1 | 1 |
| ENSMUSG00000026632 | Tatdn3 | | TatD DNase domain containing 3(Tatdn3) | 1 | 1 |
| ENSMUSG00000090165 | Ugt1a10 | | UDP glycosyltransferase 1 family, polypeptide A10(Ugt1a10) | 1 | 2 |
| ENSMUSG00000003135 | Cnot11 | | CCR4-NOT transcription complex, subunit 11(Cnot11) | 1 | 2 |
| ENSMUSG00000026605 | Cenpf | | centromere protein F(Cenpf) | 1 | 1 |
| ENSMUSG00000026432 | Avpr1b | | arginine vasopressin receptor 1B(Avpr1b) | 1 | 1 |
| ENSMUSG00000037318 | Traf3ip3 * | | TRAF3 interacting protein 3(Traf3ip3) | 1 | 1 |
| ENSMUSG00000037461 | Ints7 | | integrator complex subunit 7(Ints7) | 1 | 1 |
| ENSMUSG00000114582 | 3110040M04Rik | | RIKEN cDNA 3110040M04 gene(3110040M04Rik) | 1 | - |
| ENSMUSG00000025815 | Dhtkd1 | | dehydrogenase E1 and transketolase domain containing 1(Dhtkd1) | 2 | 10 |
| ENSMUSG00000027517 | Ankrd60 | | ankyrin repeat domain 60(Ankrd60) | 2 | 20 |
| ENSMUSG00000042404 | Dennd4b | | DENN/MADD domain containing 4B(Dennd4b) | 3 | 1 |
| ENSMUSG00000046447 | Camk2n1 | | calcium/calmodulin-dependent protein kinase II inhibitor 1(Camk2n1) | 4 | 1 |
| ENSMUSG00000005514 | Por | | cytochrome p450 oxidoreductase(Por) | 5 | 7 |
| ENSMUSG00000091897 | Gm17019 | | predicted gene 17019 (Gm17019) | 5 | - |
| ENSMUSG00000029707 | Fscn3 | | fascin actin-bundling protein 3(Fscn3) | 6 | 7 |
| ENSMUSG00000061099 | Gapdhs | | glyceraldehyde-3-phosphate dehydrogenase, spermatogenic(Gapdhs) | 7 | 19 |
| ENSMUSG00000095864 | Vmn1r77 | | vomeronasal 1 receptor 77(Vmn1r77) | 7 | - |
| ENSMUSG00000062007 | Hsh2d | | hematopoietic SH2 domain containing(Hsh2d) | 8 | 19 |
| ENSMUSG00000074064 | Mlycd | | malonyl-CoA decarboxylase(Mlycd) | 8 | 16 |
| ENSMUSG00000074564 | [Gm10720](http://www.informatics.jax.org/marker/MGI:3641687) | | Predicted gene 10720 (Gm10720) | 9 | - |
| ENSMUSG00000095891 | [Gm10717](http://www.informatics.jax.org/marker/MGI:3642031) | | Predicted gene 10717 (Gm10717) | 9 | - |
| ENSMUSG00000096385 | [Gm11168](http://www.informatics.jax.org/marker/MGI:3779420) | | Predicted gene 11168 (Gm11168) | 9 | - |
| ENSMUSG00000062075 | Lmnb2 | | lamin B2(Lmnb2) | 10 | 19 |
| ENSMUSG00000058537 | [AW822073](http://www.informatics.jax.org/marker/MGI:3034577) | | expressed sequence AW822073 | 10 | - |
| ENSMUSG00000000567 | Sox9 | | SRY (sex determining region Y)-box 9(Sox9) | 11 | 17 |
| ENSMUSG00000020283 | [Pex13](http://www.informatics.jax.org/marker/MGI:1919379) | | peroxisomal biogenesis factor 13(Pex13) | 11 | 2 |
| ENSMUSG00000040447 | Spns2 | | spinster homolog 2(Spns2) | 11 | 17 |
| ENSMUSG00000059395 | Nkapl | | NFKB activating protein-like(Nkapl) | 13 | 6 |
| ENSMUSG00000021940 | Ptpn20 | | protein tyrosine phosphatase, non-receptor type 20(Ptpn20) | 14 | 10 |
| ENSMUSG00000022091 | Sorbs3 | | sorbin and SH3 domain containing 3(Sorbs3) | 14 | 8 |
| ENSMUSG00000091477 | Gm5799 * | | predicted gene 5799 (Gm5799) | 14 | - |
| [ENSMUSG00000095280](http://asia.ensembl.org/Mus_musculus/Gene/Idhistory?g=ENSMUSG00000095280) | Gm21738 | | predicted gene 21738 (Gm21738) | 14 | - |
| ENSMUSG00000053453 | Thoc7 | | THO complex 7(Thoc7) | 14 | 3 |
| ENSMUSG00000000606 | Vmn2r88 | | vomeronasal 2, receptor 88(Vmn2r88) | 14 | - |
| ENSMUSG00000022561 | Gpaa1 | | GPI anchor attachment protein 1(Gpaa1) | 15 | 8 |
| ENSMUSG00000032796 | Lama1 | | laminin, alpha 1(Lama1) | 17 | 18 |
| ENSMUSG00000001228 | Uhrf1 | | ubiquitin-like, containing PHD and RING finger domains, 1(Uhrf1) | 17 | 19 |
| ENSMUSG00000024220 | Zfp523 | | zinc finger protein 523 (Zfp523) | 17 | 6 |
| ENSMUSG00000057000 | Nxf3 * | | nuclear RNA export factor 3(Nxf3) | X | X |
| ENSMUSG00000069036 | Sry | | sex determining region of Chr Y(Sry) | Y | Y |
| ENSMUSG00000096223 | [Gm21874](http://www.informatics.jax.org/marker/MGI:5434038) | | Predicted gene, 21874 (Gm21874) | Y | - |
| ENSMUSG00000096808 | - | |  | - |  |
| ENSMUSG00000095041 | - | |  | - |  |

- These genes mutation repertoire are only found in gefitinib-resistance EGFR^L858R^-p53^+/+^ lung cancer mice.

* These genes are both found in gefitinib-resistance EGFR^L858R^-p53^+/-^ and EGFR^L858R^-p53^+/+^ lung cancer mice.
