# Supplementary material for: Tp53 haploinsufficiency is involved in hotspot mutations and cytoskeletal remodeling in gefitinib-induced drug-resistant EGFRL858R-lung cancer mice
Source: Cell Death Discov. 2023 Mar 14;9:96. doi: 10.1038/s41420-023-01393-2 (PMC10015023; doi:10.1038/s41420-023-01393-2)
Supplement: Supplementary file 5 — Supplementary Table 3 [file 41420_2023_1393_MOESM5_ESM.docx]

**Supplementary Table 3. The tumorigenesis gene list in EGFR^L858R^-p53^+/-^ mice**

| **Ensembl Gene ID** | **Gene Name** | **Gene Full Name** | **CHROM (**mice) | **CHROM (**human) |
| --- | --- | --- | --- | --- |
| ENSMUSG00000026259 | [Ngef](http://www.ensembl.org/Search/Results?species=all;q=Ngef) | neuronal guanine nucleotide exchange factor(Ngef) | 1 | 2 |
| ENSMUSG00000061024 | [Rrs1](http://www.ensembl.org/Search/Results?species=all;q=Rrs1) | ribosome biogenesis regulator 1(Rrs1) | 1 | 8 |
| ENSMUSG00000041926 | [Rnpep](http://www.ensembl.org/Search/Results?species=all;q=Rnpep) | arginyl aminopeptidase (aminopeptidase B)(Rnpep) | 1 | 1 |
| ENSMUSG00000090145 | [Ugt1a6b](http://www.ensembl.org/Search/Results?species=all;q=Ugt1a6b) | UDP glucuronosyltransferase 1 family, polypeptide A6B(Ugt1a6b) | 1 | 2 |
| ENSMUSG00000114582 | [3110040M04Rik](http://www.ensembl.org/Search/Results?species=all;q=3110040M04Rik) | RIKEN cDNA 3110040M04 gene(3110040M04Rik) | 1 | - |
| ENSMUSG00000027242 | [Wdr76](http://www.ensembl.org/Search/Results?species=all;q=Wdr76) | WD repeat domain 76(Wdr76) | 2 | 15 |
| ENSMUSG00000034552 | [Zswim2](http://www.ensembl.org/Search/Results?species=all;q=Zswim2) | zinc finger SWIM-type containing 2(Zswim2) | 2 | 2 |
| ENSMUSG00000036587 | [Fut7](http://www.ensembl.org/Search/Results?species=all;q=Fut7) | fucosyltransferase 7(Fut7) | 2 | 9 |
| ENSMUSG00000017740 | [Slc12a5](http://www.ensembl.org/Search/Results?species=all;q=Slc12a5) | solute carrier family 12, member 5(Slc12a5) | 2 | 20 |
| ENSMUSG00000041984 | [Rptn](http://www.ensembl.org/Search/Results?species=all;q=Rptn) | repetin(Rptn) | 3 | 1 |
| ENSMUSG00000028101 | [Pias3](http://www.ensembl.org/Search/Results?species=all;q=Pias3) | protein inhibitor of activated STAT 3(Pias3) | 3 | 1 |
| ENSMUSG00000027881 | [Prpf38b](http://www.ensembl.org/Search/Results?species=all;q=Prpf38b) | PRP38 pre-mRNA processing factor 38 (yeast) domain containing B(Prpf38b) | 3 | 1 |
| ENSMUSG00000102439 | [Flg](http://www.ensembl.org/Search/Results?species=all;q=Flg) | filaggrin(Flg) | 3 | - |
| ENSMUSG00000000682 | [Cd52](http://www.ensembl.org/Search/Results?species=all;q=Cd52) | CD52 antigen(Cd52) | 4 | 1 |
| ENSMUSG00000028756 | [Pink1](http://www.ensembl.org/Search/Results?species=all;q=Pink1) | PTEN induced putative kinase 1(Pink1) | 4 | 1 |
| ENSMUSG00000042388 | [Dlgap3](http://www.ensembl.org/Search/Results?species=all;q=Dlgap3) | DLG associated protein 3(Dlgap3) | 4 | 1 |
| ENSMUSG00000115466 | [Vmn1r3](http://www.ensembl.org/Search/Results?species=all;q=Vmn1r3) | vomeronasal 1 receptor 3(Vmn1r3) | 4 | - |
| ENSMUSG00000078503 | [Zfp990](http://www.ensembl.org/Search/Results?species=all;q=Zfp990) | zinc finger protein 990(Zfp990) | 4 | - |
| ENSMUSG00000039358 | [Drd5](http://www.ensembl.org/Search/Results?species=all;q=Drd5) | dopamine receptor D5(Drd5) | 5 | 4 |
| ENSMUSG00000095550 | [Gm21671](http://www.ensembl.org/Search/Results?species=all;q=Gm21671) | predicted gene, 21671(Gm21671) | 5 | - |
| ENSMUSG00000091897 | [Gm17019](http://www.ensembl.org/Search/Results?species=all;q=Gm17019) | predicted gene 17019(Gm17019) | 5 | - |
| ENSMUSG00000067543 | [Prb1](http://www.ensembl.org/Search/Results?species=all;q=Prb1) | proline-rich protein BstNI subfamily 1(Prb1) | 6 | 12 |
| ENSMUSG00000013921 | [Clip3](http://www.ensembl.org/Search/Results?species=all;q=Clip3) | CAP-GLY domain containing linker protein 3(Clip3) | 7 | 19 |
| ENSMUSG00000015165 | [Hnrnpl](http://www.ensembl.org/Search/Results?species=all;q=Hnrnpl) | heterogeneous nuclear ribonucleoprotein L(Hnrnpl) | 7 | 19 |
| ENSMUSG00000040488 | [Ltbp4](http://www.ensembl.org/Search/Results?species=all;q=Ltbp4) | latent transforming growth factor beta binding protein 4(Ltbp4) | 7 | 19 |
| ENSMUSG00000058886 | [Deaf1](http://www.ensembl.org/Search/Results?species=all;q=Deaf1) | DEAF1, transcription factor(Deaf1) | 7 | - |
| ENSMUSG00000003484 | [Cyp4f18](http://www.ensembl.org/Search/Results?species=all;q=Cyp4f18) | cytochrome P450, family 4, subfamily f, polypeptide 18(Cyp4f18) | 8 | 19 |
| ENSMUSG00000096265 | [Potefam3e](http://www.ensembl.org/Search/Results?species=all;q=Potefam3e) | POTE ankyrin domain family member 3E(Potefam3e) | 8 | 2 |
| ENSMUSG00000041268 | [Dmxl2](http://www.ensembl.org/Search/Results?species=all;q=Dmxl2) | Dmx-like 2(Dmxl2) | 9 | 15 |
| ENSMUSG00000096201 | [Gm10715](http://www.ensembl.org/Search/Results?species=all;q=Gm10715) | Predicted gene 10715(Gm10715) | 9 | - |
| ENSMUSG00000096385 | [Gm11168](http://www.ensembl.org/Search/Results?species=all;q=Gm11168) | Predicted gene 11168(Gm11168) | 9 | - |
| ENSMUSG00000074505 | [Fat3](http://www.ensembl.org/Search/Results?species=all;q=Fat3) | FAT atypical cadherin 3(Fat3) | 9 | 11 |
| ENSMUSG00000020131 | [Pcsk4](http://www.ensembl.org/Search/Results?species=all;q=Pcsk4) | proprotein convertase subtilisin/kexin type 4(Pcsk4) | 10 | 19 |
| ENSMUSG00000058298 | [Mcm9](http://www.ensembl.org/Search/Results?species=all;q=Mcm9) | minichromosome maintenance 9 homologous recombination repair factor(Mcm9) | 10 | 6 |
| ENSMUSG00000020329 | [Polrmt](http://www.ensembl.org/Search/Results?species=all;q=Polrmt) | polymerase (RNA) mitochondrial (DNA directed)(Polrmt) | 10 | 19 |
| ENSMUSG00000055775 | [Myh8](http://www.ensembl.org/Search/Results?species=all;q=Myh8) | myosin, heavy polypeptide 8, skeletal muscle, perinatal(Myh8) | 11 | 17 |
| ENSMUSG00000042436 | [Mfap4](http://www.ensembl.org/Search/Results?species=all;q=Mfap4) | microfibrillar-associated protein 4(Mfap4) | 11 | 17 |
| ENSMUSG00000040463 | [Mybbp1a](http://www.ensembl.org/Search/Results?species=all;q=Mybbp1a) | MYB binding protein (P160) 1a(Mybbp1a) | 11 | 17 |
| ENSMUSG00000051790 | [Nlgn2](http://www.ensembl.org/Search/Results?species=all;q=Nlgn2) | neuroligin 2(Nlgn2) | 11 | 17 |
| ENSMUSG00000018340 | [Anxa6](http://www.ensembl.org/Search/Results?species=all;q=Anxa6) | annexin A6(Anxa6) | 11 | 5 |
| ENSMUSG00000079092 | [Prl2c2](http://www.ensembl.org/Search/Results?species=all;q=Prl2c2) | prolactin family 2, subfamily c, member 2(Prl2c2) | 13 | - |
| ENSMUSG00000022019 | [Tdrd3](http://www.ensembl.org/Search/Results?species=all;q=Tdrd3) | tudor domain containing 3(Tdrd3) | 14 | 8 |
| ENSMUSG00000022840 | [Adcy5](http://www.ensembl.org/Search/Results?species=all;q=Adcy5) | adenylate cyclase 5(Adcy5) | 16 | 3 |
| ENSMUSG00000022510 | [Trp63](http://www.ensembl.org/Search/Results?species=all;q=Trp63) | transformation related protein 63(Trp63) | 16 | 3 |
| ENSMUSG00000022899 | [Slc15a2](http://www.ensembl.org/Search/Results?species=all;q=Slc15a2) | solute carrier family 15 (H+/peptide transporter), member 2(Slc15a2) | 16 | 3 |
| ENSMUSG00000024392 | [Bag6](http://www.ensembl.org/Search/Results?species=all;q=Bag6) | BCL2-associated athanogene 6(Bag6) | 17 | 6 |
| ENSMUSG00000051977 | [Prdm9](http://www.ensembl.org/Search/Results?species=all;q=Prdm9) | PR domain containing 9(Prdm9) | 17 | 5, 16 |
| ENSMUSG00000069729 | [Arid1b](http://www.ensembl.org/Search/Results?species=all;q=Arid1b) | AT rich interactive domain 1B (SWI-like)(Arid1b) | 17 | 6 |
| ENSMUSG00000033323 | [Ctdp1](http://www.ensembl.org/Search/Results?species=all;q=Ctdp1) | CTD (carboxy-terminal domain, RNA polymerase II, polypeptide A) phosphatase, subunit 1(Ctdp1) | 18 | 18 |
| ENSMUSG00000024597 | [Slc12a2](http://www.ensembl.org/Search/Results?species=all;q=Slc12a2) | solute carrier family 12, member 2(Slc12a2) | 18 | 5 |
| ENSMUSG00000040414 | [Slc25a28](http://www.ensembl.org/Search/Results?species=all;q=Slc25a28) | solute carrier family 25, member 28(Slc25a28) | 19 | 10 |
| ENSMUSG00000062783 | [Csprs](http://www.ensembl.org/Search/Results?species=all;q=Csprs) | component of Sp100-rs(Csprs) | GL456221.1 | - |
| ENSMUSG00000090546 | [Cdr1](http://www.ensembl.org/Search/Results?species=all;q=Cdr1) | cerebellar degeneration related antigen 1(Cdr1) | X | X |

- These genes mutation repertoire are only found in gefitinib-sensitive EGFR^L858R^-p53^+/-^ lung cancer mice.
