# Supplementary material for: Tp53 haploinsufficiency is involved in hotspot mutations and cytoskeletal remodeling in gefitinib-induced drug-resistant EGFRL858R-lung cancer mice
Source: Cell Death Discov. 2023 Mar 14;9:96. doi: 10.1038/s41420-023-01393-2 (PMC10015023; doi:10.1038/s41420-023-01393-2)
Supplement: Supplementary file 6 — Supplementary Table 4 [file 41420_2023_1393_MOESM6_ESM.docx]

**Supplementary Table 4. The drug-resistance gene list in EGFR^L858R^-p53^+/-^ mice**

| **Ensembl Gene ID** | | **Gene Name** | **Gene Full Name** | **CHROM**（mice) | **CHROM**（human） |
| --- | --- | --- | --- | --- | --- |
| ENSMUSG00000054545 | [Ugt1a6a](http://www.ensembl.org/Search/Results?species=all;q=Ugt1a6a) | | UDP glucuronosyltransferase 1 family, polypeptide A6A(Ugt1a6a) | 1 | 2 |
| ENSMUSG00000026622 | [Nek2](http://www.ensembl.org/Search/Results?species=all;q=Nek2) | | NIMA (never in mitosis gene a)-related expressed kinase 2(Nek2) | 1 | 1 |
| ENSMUSG00000037375 | [Hhat](http://www.ensembl.org/Search/Results?species=all;q=Hhat) * | | hedgehog acyltransferase(Hhat) | 1 | 1 |
| ENSMUSG00000058248 | [Kcnh1](http://www.ensembl.org/Search/Results?species=all;q=Kcnh1) * | | potassium voltage-gated channel, subfamily H (eag-related), member 1(Kcnh1) | 1 | 1 |
| ENSMUSG00000026579 | [F5](http://www.ensembl.org/Search/Results?species=all;q=F5) | | coagulation factor V(F5) | 1 | 1 |
| ENSMUSG00000037318 | [Traf3ip3](http://www.ensembl.org/Search/Results?species=all;q=Traf3ip3) * | | TRAF3 interacting protein 3(Traf3ip3) | 1 | 1 |
| ENSMUSG00000006014 | [Prg4](http://www.ensembl.org/Search/Results?species=all;q=Prg4) | | proteoglycan 4 (megakaryocyte stimulating factor, articular superficial zone protein)(Prg4) | 1 | 1 |
| ENSMUSG00000114591 | [Gm47996](http://www.ensembl.org/Search/Results?species=all;q=Gm47996) | | predicted gene, 47996(Gm47996) | 1 | 15 |
| ENSMUSG00000027254 | [Map1a](http://www.ensembl.org/Search/Results?species=all;q=Map1a) | | microtubule-associated protein 1 A(Map1a) | 2 | 15 |
| ENSMUSG00000095028 | [Sirpb1b](http://www.ensembl.org/Search/Results?species=all;q=Sirpb1b) | | signal-regulatory protein beta 1B(Sirpb1b) | 3 | 20 |
| ENSMUSG00000039701 | [Usp53](http://www.ensembl.org/Search/Results?species=all;q=Usp53) | | ubiquitin specific peptidase 53(Usp53) | 3 | 4 |
| ENSMUSG00000050213 | [Snip1](http://www.ensembl.org/Search/Results?species=all;q=Snip1) | | Smad nuclear interacting protein 1(Snip1) | 4 | 1 |
| ENSMUSG00000050966 | [Lin28a](http://www.ensembl.org/Search/Results?species=all;q=Lin28a) | | lin-28 homolog A(Lin28a) | 4 | 1 |
| ENSMUSG00000073208 | [Speer4c](http://www.ensembl.org/Search/Results?species=all;q=Speer4c) | | spermatogenesis associated glutamate (E)-rich protein 4C(Speer4c) | 5 | - |
| ENSMUSG00000094282 | [Cfap73](http://www.ensembl.org/Search/Results?species=all;q=Cfap73) | | cilia and flagella associated protein 73(Cfap73) | 5 | 12 |
| ENSMUSG00000030329 | [Pianp](http://www.ensembl.org/Search/Results?species=all;q=Pianp) | | PILR alpha associated neural protein(Pianp) | 6 | 12 |
| ENSMUSG00000051586 | [Mical3](http://www.ensembl.org/Search/Results?species=all;q=Mical3) | | microtubule associated monooxygenase, calponin and LIM domain containing 3(Mical3) | 6 | 22 |
| ENSMUSG00000032637 | [Atxn2l](http://www.ensembl.org/Search/Results?species=all;q=Atxn2l) | | ataxin 2-like(Atxn2l) | 7 | 16 |
| ENSMUSG00000031805 | [Jak3](http://www.ensembl.org/Search/Results?species=all;q=Jak3) | | Janus kinase 3(Jak3) | 8 | 19 |
| ENSMUSG00000048827 | [Pkd1l3](http://www.ensembl.org/Search/Results?species=all;q=Pkd1l3) | | polycystic kidney disease 1 like 3(Pkd1l3) | 8 | 16 |
| ENSMUSG00000035569 | [Ankrd11](http://www.ensembl.org/Search/Results?species=all;q=Ankrd11) | | ankyrin repeat domain 11(Ankrd11) | 8 | 16 |
| ENSMUSG00000031788 | [Kifc3](http://www.ensembl.org/Search/Results?species=all;q=Kifc3) | | kinesin family member C3(Kifc3) | 8 | 16 |
| ENSMUSG00000025243 | [Slc6a20b](http://www.ensembl.org/Search/Results?species=all;q=Slc6a20b) | | solute carrier family 6 (neurotransmitter transporter), member 20B(Slc6a20b) | 9 | 3 |
| ENSMUSG00000047237 | [Fbxw21](http://www.ensembl.org/Search/Results?species=all;q=Fbxw21) | | F-box and WD-40 domain protein 21(Fbxw21) | 9 | 3 |
| ENSMUSG00000074564 | [Gm10720](http://www.ensembl.org/Search/Results?species=all;q=Gm10720) | | Predicted gene 10720(Gm10720) | 9 | - |
| ENSMUSG00000090592 | [Gm17571](http://www.ensembl.org/Search/Results?species=all;q=Gm17571) | | Predicted gene, 17571(Gm17571) | 9 | - |
| ENSMUSG00000095547 | [Gm10719](http://www.ensembl.org/Search/Results?species=all;q=Gm10719) | | Predicted gene 10719(Gm10719) | 9 | - |
| ENSMUSG00000034684 | [Sema3f](http://www.ensembl.org/Search/Results?species=all;q=Sema3f) | | sema domain, immunoglobulin domain (Ig), short basic domain, secreted, (semaphorin) 3F(Sema3f) | 9 | 3 |
| ENSMUSG00000019979 | [Apaf1](http://www.ensembl.org/Search/Results?species=all;q=Apaf1) | | apoptotic peptidase activating factor 1(Apaf1) | 10 | 12 |
| ENSMUSG00000004668 | [Abca13](http://www.ensembl.org/Search/Results?species=all;q=Abca13) | | ATP-binding cassette, sub-family A (ABC1), member 13(Abca13) | 11 | 7 |
| ENSMUSG00000009210 | [Prr29](http://www.ensembl.org/Search/Results?species=all;q=Prr29) | | proline rich 29(Prr29) | 11 | 17 |
| ENSMUSG00000033987 | [Dnah17](http://www.ensembl.org/Search/Results?species=all;q=Dnah17) | | dynein, axonemal, heavy chain 17(Dnah17) | 11 | 17 |
| ENSMUSG00000050545 | [Fam228b](http://www.ensembl.org/Search/Results?species=all;q=Fam228b) | | family with sequence similarity 228, member B(Fam228b) | 12 | 2 |
| ENSMUSG00000047419 | [Cmya5](http://www.ensembl.org/Search/Results?species=all;q=Cmya5) | | cardiomyopathy associated 5(Cmya5) | 13 | 5 |
| ENSMUSG00000001504 | [Irx2](http://www.ensembl.org/Search/Results?species=all;q=Irx2) | | Iroquois homeobox 2(Irx2) | 13 | 5 |
| ENSMUSG00000091347 | [Gm10772](http://www.ensembl.org/Search/Results?species=all;q=Gm10772) | | predicted gene 10772(Gm10772) | 13 | - |
| ENSMUSG00000070448 | [Vmn2r89](http://www.ensembl.org/Search/Results?species=all;q=Vmn2r89) | | vomeronasal 2, receptor 89(Vmn2r89) | 14 | - |
| ENSMUSG00000091477 | [Gm5799](http://www.ensembl.org/Search/Results?species=all;q=Gm5799) * | | predicted gene 5799(Gm5799) | 14 | - |
| ENSMUSG00000021843 | [Ktn1](http://www.ensembl.org/Search/Results?species=all;q=Ktn1) | | kinectin 1(Ktn1) | 14 | 14 |
| ENSMUSG00000055737 | [Ghr](http://www.ensembl.org/Search/Results?species=all;q=Ghr) | | growth hormone receptor(Ghr) | 15 | 5 |
| ENSMUSG00000033565 | [Rbfox2](http://www.ensembl.org/Search/Results?species=all;q=Rbfox2) | | RNA binding protein, fox-1 homolog (C. elegans) 2(Rbfox2) | 15 | 22 |
| ENSMUSG00000005836 | [Gata6](http://www.ensembl.org/Search/Results?species=all;q=Gata6) | | GATA binding protein 6(Gata6) | 18 | 18 |
| ENSMUSG00000024913 | [Lrp5](http://www.ensembl.org/Search/Results?species=all;q=Lrp5) | | low density lipoprotein receptor-related protein 5(Lrp5) | 19 | 11, 22 |
| ENSMUSG00000034765 | [Dusp5](http://www.ensembl.org/Search/Results?species=all;q=Dusp5) | | dual specificity phosphatase 5(Dusp5) | 19 | 10 |
| ENSMUSG00000057000 | [Nxf3](http://www.ensembl.org/Search/Results?species=all;q=Nxf3) * | | nuclear RNA export factor 3(Nxf3) | X | X |
| ENSMUSG00000072049 | [Vmn2r121](http://www.ensembl.org/Search/Results?species=all;q=Vmn2r121) | | vomeronasal 2, receptor 121(Vmn2r121) | X | - |

- These genes mutation repertoire are only found in gefitinib-resistance EGFR^L858R^-p53^+/-^ lung cancer mice.

* These genes are both found in gefitinib-resistance EGFR^L858R^-p53^+/-^ and EGFR^L858R^-p53^+/+^ lung cancer mice.
