# Supplementary material for: Tp53 haploinsufficiency is involved in hotspot mutations and cytoskeletal remodeling in gefitinib-induced drug-resistant EGFRL858R-lung cancer mice
Source: Cell Death Discov. 2023 Mar 14;9:96. doi: 10.1038/s41420-023-01393-2 (PMC10015023; doi:10.1038/s41420-023-01393-2)
Supplement: Supplementary file 7 — Supplementary Table 5 [file 41420_2023_1393_MOESM7_ESM.docx]

Suppl.Table 5A. 24 genes of 53 genes mutation in drug resistant mice were also mutated in clinical lung cancer patients, which the WGS from TCGA. (201: control mice; 208: tumor induced mice; 224: drug resistant mice; red: upregulation; blue: downregulation)

Suppl.Table 5B. 11 genes of 24 genes mutation were highly mutated in clinical lung cancer patients, which the WGS from TCGA.
