# Supplementary material for: Tp53 haploinsufficiency is involved in hotspot mutations and cytoskeletal remodeling in gefitinib-induced drug-resistant EGFRL858R-lung cancer mice
Source: Cell Death Discov. 2023 Mar 14;9:96. doi: 10.1038/s41420-023-01393-2 (PMC10015023; doi:10.1038/s41420-023-01393-2)
Supplement: Supplementary file 8 — Supplementary Table 6 [file 41420_2023_1393_MOESM8_ESM.docx]

Suppl.Table 6. Genes regulated during drug resistance (pink: up-regulation; blue: down-regulation; RO201L: control mice; Dox208L: tumor induced mice; DoxGe224R/DoxgE1185L/DoxGe1185R: drug resistant mice)
