# Supplementary material for: Tp53 haploinsufficiency is involved in hotspot mutations and cytoskeletal remodeling in gefitinib-induced drug-resistant EGFRL858R-lung cancer mice
Source: Cell Death Discov. 2023 Mar 14;9:96. doi: 10.1038/s41420-023-01393-2 (PMC10015023; doi:10.1038/s41420-023-01393-2)
Supplement: Supplementary file 9 — Supplementary Table 7 [file 41420_2023_1393_MOESM9_ESM.docx]

Suppl. Table 7. All the sequences of primers used in this study

| Name | Primer sequence |
| --- | --- |
| CTBP1 (human) | Forward: 5’- CCGTCAAGCAGATGAGACAA-3’ |
|  | Reverse: 5’- GGCTAAAGCTGAAGGGTTCC-3’ |
| Ctbp1 (mouse) | Forward: 5’-CTGCACAGTGGAGATGCCTA -3’ |
|  | Reverse: 5’- CCAATTCGGACGATGATTCT-3’ |
| TNNT3 (human) | Forward: 5’- GATCCCAGAAGGGGAGAAAG-3’ |
|  | Reverse: 5’- CCTTCTCTGCACGAATCCTC -3’ |
| Tnnt3 (mouse) | Forward: 5’- ATGCCAAGAGGAGAGCTGAA-3’ |
|  | Reverse: 5’- CAGAGTTCCTTGGCCTTGTC-3’ |
| Myh1 (mouse) | Forward: 5’- AGAGCCAAGAGGAAACTGGAGG -3’ |
|  | Reverse: 5’- CTCGTCCTCAATCTTGCTCTGC -3’ |
| MYH4 (human) | Forward: 5’- CCTGGAACGGACTGAGAGAG-3’ |
|  | Reverse: 5’- TCCATCTCTCCCTGGATTTG-3’ |
| Myh4 (mouse) | Forward: 5’- CAAGTCATCGGTGTTTGTGG-3’ |
|  | Reverse: 5’- TGTCGTACTTGGGAGGGTTC-3’ |
| MYH8 (human) | Forward: 5’- CCGTCAAGCAGATGAGACAA-3’ |
|  | Reverse: 5’- GGCTAAAGCTGAAGGGTTCC-3’ |
| Myh8 (mouse) | Forward: 5’- GGCCAAAATCAAAGAGGTGA-3’ |
|  | Reverse: 5’- CGTGCTTCTCCTTCTCAACC-3’ |
| Kif6 (mouse) | Forward: 5’- TCGGAAAAACACCGTACACA-3’ |
|  | Reverse: 5’- CTTTTGCAAGCGAACAATCA-3’ |
| Tpx2 (mouse) | Forward: 5’- GGCAAGAAATGTGGAGGTGT-3’ |
|  | Reverse: 5’- TGGCAGGTTAATGGTGTCAA-3’ |
| Mefv (mouse) | Forward: 5’- AGAGAAGGAGAACCCCCAAA-3’ |
|  | Reverse: 5’- CATCTCTCCTCCCCAATCAA-3’ |
| Tubb3 (mouse) | Forward: 5’- TGAGGCCTCCTCTCACAAGT-3’ |
|  | Reverse: 5’- CGCACGACATCTAGGACTGA-3’ |

| **Name** | **Primer sequence** |
| --- | --- |
| CTBP1 (human) | Forward: 5’- CCGTCAAGCAGATGAGACAA-3’ |
|  | Reverse: 5’- GGCTAAAGCTGAAGGGTTCC-3’ |
| Ctbp1 (mouse) | Forward: 5’-CTGCACAGTGGAGATGCCTA -3’ |
|  | Reverse: 5’- CCAATTCGGACGATGATTCT-3’ |
| TNNT3 (human) | Forward: 5’- GATCCCAGAAGGGGAGAAAG-3’ |
|  | Reverse: 5’- CCTTCTCTGCACGAATCCTC -3’ |
| Tnnt3 (mouse) | Forward: 5’- ATGCCAAGAGGAGAGCTGAA-3’ |
|  | Reverse: 5’- CAGAGTTCCTTGGCCTTGTC-3’ |
| Myh1 (mouse) | Forward: 5’- AGAGCCAAGAGGAAACTGGAGG -3’ |
|  | Reverse: 5’- CTCGTCCTCAATCTTGCTCTGC -3’ |
| MYH4 (human) | Forward: 5’- CCTGGAACGGACTGAGAGAG-3’ |
|  | Reverse: 5’- TCCATCTCTCCCTGGATTTG-3’ |
| Myh4 (mouse) | Forward: 5’- CAAGTCATCGGTGTTTGTGG-3’ |
|  | Reverse: 5’- TGTCGTACTTGGGAGGGTTC-3’ |
| MYH8 (human) | Forward: 5’- CCGTCAAGCAGATGAGACAA-3’ |
|  | Reverse: 5’- GGCTAAAGCTGAAGGGTTCC-3’ |
| Myh8 (mouse) | Forward: 5’- GGCCAAAATCAAAGAGGTGA-3’ |
|  | Reverse: 5’- CGTGCTTCTCCTTCTCAACC-3’ |
| Kif6 (mouse) | Forward: 5’- TCGGAAAAACACCGTACACA-3’ |
|  | Reverse: 5’- CTTTTGCAAGCGAACAATCA-3’ |
| Tpx2 (mouse) | Forward: 5’- GGCAAGAAATGTGGAGGTGT-3’ |
|  | Reverse: 5’- TGGCAGGTTAATGGTGTCAA-3’ |
| Mefv (mouse) | Forward: 5’- AGAGAAGGAGAACCCCCAAA-3’ |
|  | Reverse: 5’- CATCTCTCCTCCCCAATCAA-3’ |
| Tubb3 (mouse) | Forward: 5’- TGAGGCCTCCTCTCACAAGT-3’ |
|  | Reverse: 5’- CGCACGACATCTAGGACTGA-3’ |
