# Supplementary material for: Tp53 haploinsufficiency is involved in hotspot mutations and cytoskeletal remodeling in gefitinib-induced drug-resistant EGFRL858R-lung cancer mice
Source: Cell Death Discov. 2023 Mar 14;9:96. doi: 10.1038/s41420-023-01393-2 (PMC10015023; doi:10.1038/s41420-023-01393-2)
Supplement: Supplementary file 10 — Supplementary material and methods [file 41420_2023_1393_MOESM10_ESM.docx]

Supplementary material and methods

**H&E and Mason’s staining** - The lung tissues from all mice were collected, routinely fixed in 4% formalin at 4˚C for 48 h and embedded in paraffin. Sections of 5‑µm thickness were cut then deparaffinized. Hematoxylin and eosin (H&E) staining was performed to observe histological changes. Briefly, the lung sections were stained with eosin for 30 secs at room temperature, rinsed with running water for 10 min and then dyed with hematoxylin for 5 min at room temperature. Masson's trichrome staining was performed using Masson's trichrome kits (Abcam, ab150686) to measure the density of collagen fibers according to the manufacturer' instruction.

**RNA-Seq.** - The RNAs were subjected to the SimpliNano™-Biochrom Spectrophotometer (Biochrom, Holliston, MA, USA) for purity and quantity and the Qsep 100 DNA/RNA Analyzer (BiOptic Inc., New Taipei City, Taiwan) for integrity, respectively. RNA fragmentation and library preparation (KAPA Biosystems, Roche, Basel, Switzerland) were carried out by the NovaSeq 6000 System (Illumina, San Diego, CA, USA), through which the constructed libraries were analyzed by 150 bp paired-end high-throughput sequencing at Biotools Co., Ltd. (New Taipei City, Taiwan). Briefly, read pairs mapping from each sample were aligned to the reference genome (i.e., GRCh38) by the HISAT2 software (v2.1.0) and the results were reported following the “fragments per kilobase of transcript per million mapped reads” method, known as FPKM, which quantile normalized all samples. Genes with fragments per kilobase of transcript per million mapped reads (FPKM) values larger than four and that exhibited low variation across three biological replicates were considered reliable and were used in subsequent analyses ([Supplementary Fig. 2](https://www.sciencedirect.com/science/article/pii/S0147651321009155#sec0115)). Identification of differentially expressed genes (DEGs) among four groups was performed using the DESeq R package v1.12.0. The significance of the gene expression difference was indicated by an adjusted P < 0.05. Finally, DEGs were annotated using databases such as NCBI, Gene Ontology (GO), and Kyoto Encyclopedia of Genes and Genomes (KEGG) to obtain detailed information. Technical support of the above experiments was provided by Novogene (Beijing, China).

**Immunofluorescent analysis** - Scramble and shUSP24-knockdown lentivirus infected cells were seeded in 6-well plates with cover slips inside for 48 hours. Cover slips were removed and cells were fixed with 4% paraformaldehyde in 4°C for 15 minutes. After fixation, cover slips were washed with PBS, and incubated with 0.2% Triton X-100 in PBS for 5 minutes at room temperature. Cover slips were then blocked with 1% Bovine serum albumin (BSA) for 1 hour, and stained with anti-tubulin antibody (1:200, Genetex) or anti-actin (abcam) for 16 hours at 4°C. After washing with PBS, cells were stained with Alexa Fluor® 488 or 568 (Invitrogen) for 1h at room temperature, and mounted with 90% glycerol containing DAPI (Invitrogen).

**Whole genome sequencing** – DNA degradation and contamination were monitored on 1% agarose gels and DNA concentration was measured using Qubit® DNA Assay Kit in Qubit® 2.0 Flurometer (Life Technologies, CA, USA). A total amount of 1.0μg DNA per sample was used as input material for the DNA sample preparations. Sequencing libraries were generated using NEBNext® DNA Library Prep Kit following manufacturer's recommendations and indices were added to each sample. The genomic DNA was randomly fragmented to a size of 350bp by shearing, then DNA fragments were end polished, A-tailed, and ligated with the NEBNext adapter for Illumina sequencing, and further PCR enriched by P5 and indexed P7 oligos. The PCR products were purified (AMPure XP system) and resulted libraries were analyzed for size distribution by Agilent 2100 Bioanalyzer and quantified using real-time PCR.

Supplementary Fig. 1. Quality control of whole-genome sequencing (WGS) data in drug-sensitive mice, drug-resistant mice and mice with TP53^+/-^-mediated drug resistance. DNA was isolated from the lungs for WGS. The error rate distribution along the reads (A), sequencing depth (B), quality score distribution along the reads (C), and classification of raw reads (D) are shown here.

Supplementary Fig. 2. Quality control of RNA-seq data in drug-sensitive mice, drug-resistant mice and mice with TP53^+/-^-mediated drug resistance. Total RNAs was isolated from the lungs for RNA-seq. The error rate distribution along the reads (A), base content distribution (B), and Trimmomatic results (C) are shown here.

Supplementary Table 1. Detailed list of genes mutated in tumorigenesis (drug-sensitive mice)

Supplementary Table 2. Detailed list of genes mutated in drug resistance – 53 genes related drug resistance are listed here, and 11 genes of these genes (highlighted) were used for the further analysis shown in Fig. 3C.

Supplementary Table 3. Detailed list of genes mutated in mice with TP53^+/-^ drug sensitive.

Supplementary Table 4. Detailed list of genes mutated in a p53-dependent manner during drug resistance acquisition

Supplementary Table 5. List of the 24 genes list in TCGA cohorts with the same mutations found in drug-resistant mice. Eleven of the 24 genes had a higher mutation rate in clinical lung cancer patients.

Supplementary Table 6. Detailed profile of gene regulation in mice with gefitinib-induced drug resistant.

Supplementary Table 7. All the sequences of primers used in this study
